# Comparative Study of Ni(II) Complexes with Dithiocarbazate- and Thiosemicarbazone-Based Ligands: Synthesis, Crystal Structures, and Anticancer Activity

**DOI:** 10.3390/molecules30173516

**Published:** 2025-08-28

**Authors:** Gabriel S. Pessoa, Mariana P. Viana, Katia M. Oliveira, Claudia C. Gatto

**Affiliations:** Laboratory of Inorganic Synthesis and Crystallography, Institute of Chemistry, University of Brasilia, Brasília 70904-970, DF, Brazil

**Keywords:** Ni(II) complexes, dithiocarbazate, thiosemicarbazone, crystal structures, Hirshfeld surface, anticancer activity

## Abstract

The present work reports a comparative study of thiosemicarbazone (H_2_L^1^) and dithiocarbazate (H_2_L^2^) ligands and their Ni(II) complexes; [Ni(L^1^)(PPh_3_)] (**1**); [Ni(L^1^)(Py)] (**2**); [Ni(L^2^)(PPh_3_)] (**3**); and [Ni(L^2^)(Py)] (**4**). All compounds were characterized by spectroscopy analysis; and the complexes were also characterized by single-crystal X-ray diffraction. The crystal structures of the complexes revealed a distorted square planar geometry with the Ni(II) atoms coordinated to a double-deprotonated and tridentate ligand molecule by the *ONS* donor system. The coordination sphere is completed by the incorporation of pyridine or triphenylphosphine coligands at the metal center. Biological assays were conducted against the cell lines breast cancer (MCF-7); cisplatin-resistant ovarian cancer (A2780cis); lung cancer (A549); and nontumoral lung (MRC-5). The results show that cytotoxicity was significantly enhanced upon complexation for complexes (**2**) and (**4**); whereas it was suppressed for complexes (**1**) and (**3**) against the A2780cis and A549 cell lines. Notably; complex (**2**) exhibited superior cytotoxicity compared to cisplatin against both MCF-7 and A2780cis.

## 1. Introduction

An ideal drug should treat a disease without causing undesirable side effects, be easy to use, and be affordable. However, many of the available drugs, especially those used to treat cancer, often cause significant discomfort during administration and may have adverse side effects [1,2]. This reality highlights the importance of increasing efforts to develop new drugs and therapeutic alternatives.

**Figure 1:**
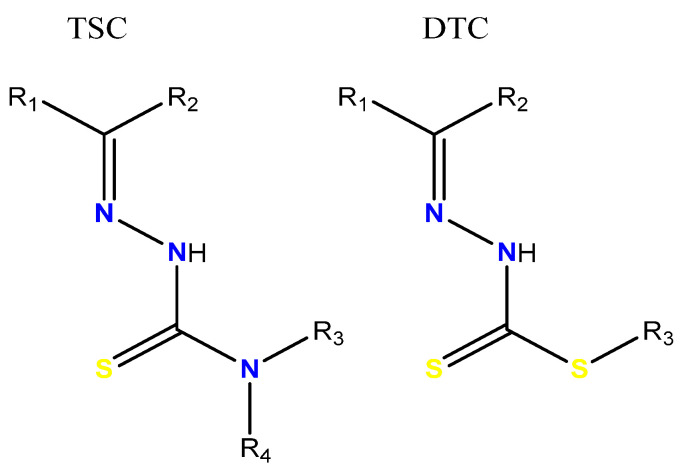
General structures of a thiosemicarbazone (TSC) and a dithiocarbazate (DTC); R = alkyl or aryl groups.

In recent decades, Schiff bases have attracted great attention due to their wide range of potential applications in various fields, such as catalysis, chemical sensors, and therapeutic agents [3,4]. Metal complexes with Schiff bases have demonstrated significant biological activity, including anticancer, antibacterial, and antifungal properties [5,6,7]. Among the known Schiff bases, thiosemicarbazones and dithiocarbazates stand out, which are characterized by their imine or azomethine functional group, with significant pharmacological and structural properties (Figure 1).

Several compounds of the thiosemicarbazone class have shown significant antitumor activity, being capable of inhibiting the growth of cancer cells [8]. Recent work demonstrates that thiosemicarbazones can coordinate with essential metal ions, such as copper and iron, disrupting tumor cell metabolism [9,10]. In addition, some thiosemicarbazones exhibit antibacterial and antiviral properties, further expanding their therapeutic potential [11].

Dithiocarbazates have been widely studied due to their structural versatility and physicochemical properties, producing metal complexes with diverse geometries and biological activities [12,13,14,15]. A variety of complexes with different metals have been described in the literature, demonstrating that the pharmacological activity of free dithiocarbazates increased after complexation against several strains of cancer cells and pathogenic microorganisms, including bacteria and fungi [13,14,16,17,18].

Nickel(II) complexes have demonstrated significant potential in medicinal chemistry due to their biological applications. Studies have shown that nickel(II) complexes can act as therapeutic agents against several diseases, including microbial infections, cancer, and leukemia. These complexes can exhibit antioxidant, anti-inflammatory, antimicrobial, and even cytotoxic activities [19,20,21,22]. Notably, nickel(II) complexes have been investigated for their interactions with SARS-CoV-2 and HIV pathogens, suggesting their potential as anti-COVID-19 and anti-HIV agents [23]. Furthermore, these complexes can show cytotoxic effects on tumor and leukemic cells, leading the scientific community to explore the mechanisms involved [24]. Overall, nickel(II) complexes offer a multifaceted approach to disease treatment, providing promising alternatives for combating a wide range of pathologies. Some published studies exploring the trifluoromethyl group on structures have demonstrated that CF_3_ substitution can influence a compound’s bioactivity. The potential enhancement of biological activity in these molecules can be attributed to increased stability and lipophilicity, making it highly relevant to pharmaceutical chemistry [25,26].

Due to our interest in metal complexes with thiosemicarbazones and dithiocarbazates, we synthesized four new Ni(II) complexes to compare in order to better understand how some structural modifications can influence their physicochemical and pharmacological properties [17,18,27,28]. In this study, we describe the synthesis and characterization by single-crystal X-ray diffraction, Hirshfeld surface, FT-–IR, UV–Vis, mass spectrometry, and ^1^H, ^19^F, and ^31^P nuclear magnetic resonance of thenoyltrifluoroacetone–4–phenyl–3–thiosemicarbazone (H_2_L^1^) and thenoyltrifluoroacetone–S–benzyldithiocarbazate (H_2_L^2^) ligands and their Ni(II) complexes. Furthermore, the biological activity of the compounds was evaluated against three cancer cell lines and one healthy cell line.

## 2. Results and Discussion

**Figure 2:**
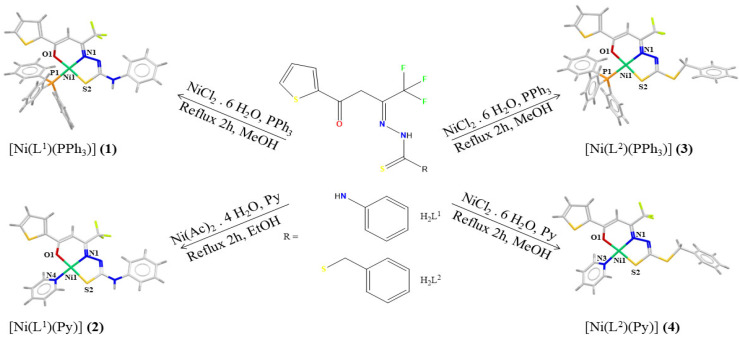
Synthesis of the complexes (**1**–**4**) obtained from the reactions between Ni(II), coligands (PPh_3_ or Py), and the ligands H_2_L^1^ and H_2_L^2^.

The complexation reactions of the H_2_L^1^ and H_2_L^2^ ligands with Ni(II) salts yielded the complexes [Ni(L^1^)(PPh_3_)] (**1**), [Ni(L^1^)(Py)] (**2**), [Ni(L^2^)(PPh_3_)] (**3**), and [Ni(L^2^)(Py)] (**4**), with triphenylphosphine or pyridine as coligand to complete the coordination sphere (Figure 2). Crystal structures of (**1**–**4**) were established by single-crystal X-ray diffraction, and physicochemical and spectroscopic analyses were performed for all compounds.

### 2.1. Structural Analyses

**Figure 3:**
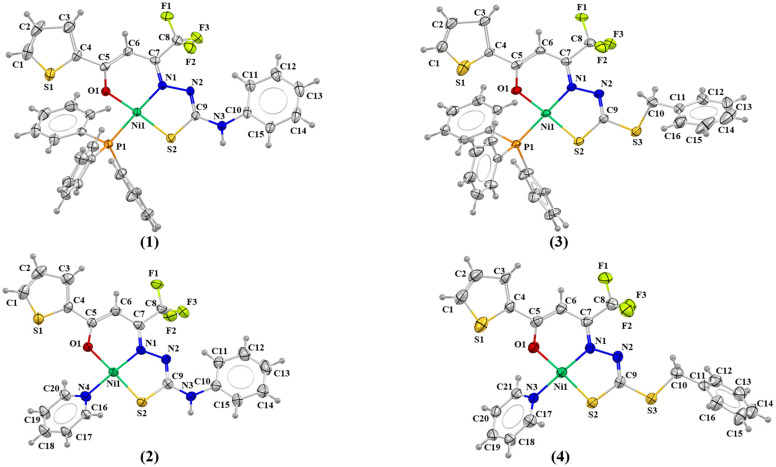
Molecular structures of the complexes (**1**–**4**) with crystallographic labeling. Thermal ellipsoids with 30% probability.

The structural analysis by single-crystal X-ray diffraction of the complexes (**1**–**4**) revealed the ligand molecules in anionic form coordinated by azomethine nitrogen, carbonyl oxygen, and thiolate sulfur atoms. The coordination sphere for the Ni(II) atoms is completed by triphenylphosphine or pyridine molecules (Figure 3). The coordination geometry proposed for all complexes is a distorted square planar arrangement with trans angles ranging between 174.23(10)° and 178.12(8)°. The Okuniewski parameter [29] (τ4) was used to verify the proposed geometry. When this parameter approaches 0, the geometry is consistent with a square arrangement, whereas values near 1 indicate a tetrahedral configuration. For all complexes, the parameter values ranged between 0.0166 and 0.0221, consistent with the observed distorted square planar geometries and aligning with similar structures [30,31]. Selected bond lengths and angles of the complexes (**1**–**4**) are listed in Table 1.

The bond lengths for the Ni1–O1 coordination were found to range between 1.854(7) and 1.835(4) Å, while Ni1–N1 bond lengths varied from 1.898(3) to 1.854(8) Å. The bond lengths of Ni1–P for triphenylphosphine are 2.204(10) and 2.209(12) Å for (**1**) and (**3**), respectively. The bond lengths of Ni1–N for pyridine are 1.925(9) and 1.903(4) Å for (**2**) and (**4**), respectively. The thiosemicarbazone and dithiocarbazate ligands are coordinated by their *E* isomer and thiol tautomer, as suggested by the bond lengths between 2.112(10) and 2.142(3) Å. Bond length values are consistent with those reported for Ni(II) complexes in the literature [17,18,30]. This is evidenced also by the bond lengths of N2–C9, which range between 1.288(5) and 1.307(13) Å, indicating a double-bond character. These values align with those observed in Schiff bases derived from other β-diketones, which exhibit N2–C9 bond lengths between 1.352(2) and 1.337(13) Å. Moreover, the C9–S2 bond lengths, which range from 1.735(6) to 1.733(3) Å, display a single-bond character. Comparatively, free Schiff base ligands derived from β-diketones exhibit shorter C9–S2 bond lengths, typically ranging from 1.677(19) to 1.645(8) Å, as reported in other works. This elongation of the C9–S2 bond in the complexes further supports their thiol tautomeric coordination [17,32,33]. The crystallographic data also revealed several non-classical intra- and intermolecular hydrogen interactions in the complexes (**1**–**4**). These interactions contribute to the stabilization of the crystal arrangement of the synthesized complexes, as shown in Table S1 and Figures S1–S4 in the Supplementary Materials. The most observed interactions present in complexes (**1**–**4**) are non-classical C–H⋯H hydrogen interactions, with distances ranging from 2.381 Å to 2.887 Å. Additionally, classical N⋯H and F⋯H interactions are present in the crystal lattice.

### 2.2. Hirshfeld Surface

**Figure 4:**
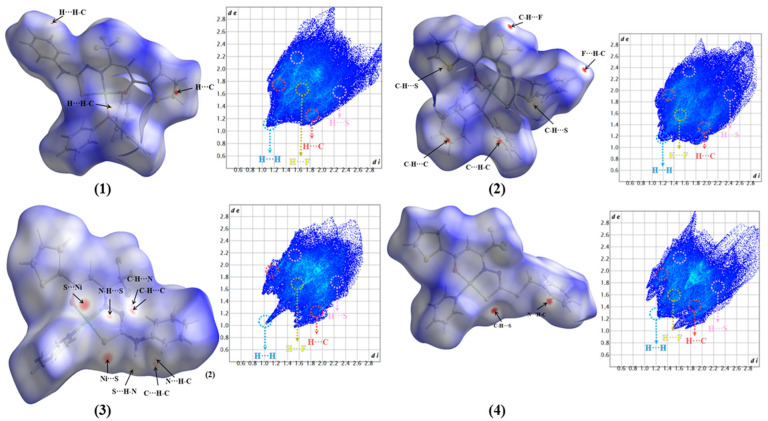
Hirshfeld surface in the d_norm_ function and a full two-dimensional fingerprint plot showing the non-covalent interactions for the complexes (**1**–**4**).

The Hirshfeld surface (HS) was used to understand and quantify the interactions in the crystal lattice packing of the complexes (**1**–**4**). HS in the d_norm_ function generates a surface that mathematically correlates the distance between an atom within the surface and the closest external atom based on van der Waals radii. This correlation produces a color pattern, ranging from blue to red, which indicates whether the contact distance is bigger, closer, or smaller than the sum of the van der Waals radii of the atoms inside the surface, respectively [34,35]. The contacts observed on the surfaces of all four complexes reveal the presence of non-classical hydrogen bonds between atoms (Figure 4). The most frequent intermolecular interactions found in the crystal structures of the complexes were between C–H⋯H–C, C–H⋯C, C–H⋯F, C–H⋯S, and C–H⋯N.

**Figure 5:**
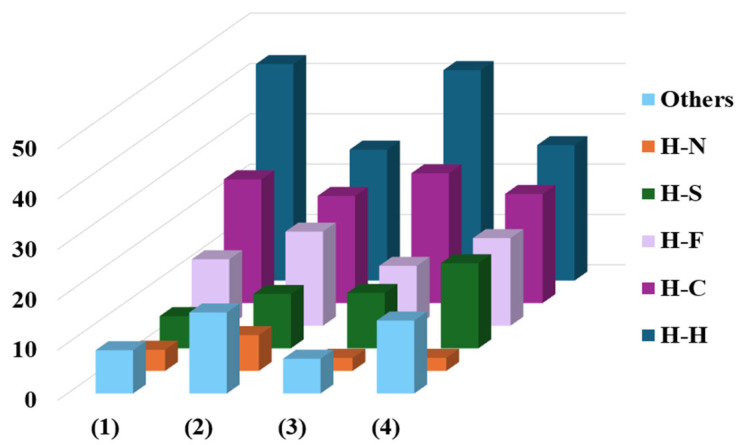
Percentage contribution of close contacts of the complexes (**1**–**4**).

The fingerprint plots of complexes (**1**–**4**) detail the interactions that most contribute to the formation of the crystal lattice, with the main contact contributions being between H⋯H and C⋯H atoms, presented in the Supplementary Materials (Figures S5–S8) and summarized in Figure 5. The H⋯H interactions contribute at 26% and 26.9% in complexes (**2**) and (**4**), respectively, significantly lower than the contributions observed in complexes (**1**) and (**3**) at 43% and 43.8%. This difference can be attributed to the presence of triphenylphosphine molecules coordinated to the metal centers. Furthermore, complexes (**1**) and (**2**) exhibit a higher contribution of H⋯N interactions, most likely from the terminal thiosemicarbazone portion in the structures, with contributions ranging between 4.2% and 7.1%. Qualitative and quantitative comparisons of the contacts present in complexes (**1**–**4**) with similar structures reported in the literature show that these contacts are frequently observed in the supramolecular arrangement of Schiff base-derived complexes [15,17,18,28,30,36,37].

### 2.3. Infrared Spectra

Infrared spectra were obtained for the ligands and their complexes, ranging from 4000 to 400 cm^−1^ (Table 2 and Figures S9–S14).

The IR spectrum of the free ligands showed the ν(N–H) of the nitrogen adjacent to the azomethine group at 3333 and 3304 cm^−1^ for H_2_L^1^ and 3195 cm^−1^ for H_2_L^2^. Additionally, both ligands exhibited a characteristic ν(C=O) at 1651 cm^−1^ for H_2_L^1^ and 1637 cm^−1^ for H_2_L^2^ [17,30,32,33,38]. Comparing the spectra of the free ligands and their Ni(II) complexes, the disappearance of the ν(C=O) and ν(N–H) bands was observed, indicating the formation of the enol tautomer with the complexation. In the absence of water molecules in complexes (**2**) and (**4**), the bands observed from 3450 to 3438 cm^−1^ can be attributed to moisture adsorbed by the KBr pellets during sample preparation. Another significant observation is the absence of the intense ν(C=S) in the complexes. This absence is attributed to the coordination of the sulfur atom with the Ni(II) atoms, which transforms this bond into a single bond, as corroborated by single-crystal X-ray diffraction results. This behavior is in agreement with the literature, where the sulfur atoms of the ligands transfer part of their electron density to the metal upon complexation, weakening the sulfur–carbon bond [15,17,27,30,32,38].

In the free H_2_L^1^ ligand, the ν(C=N) is observed at 1597 cm^−1^, while in both complexes (**1**) and (**2**) it is at 1544 cm^−1^. This shift is justified by the increased electron delocalization in the structures of the metal complexes, as supported by single-crystal X-ray diffraction results, which show the double-bond character of the C=N bond [17,28,39]. For ν(N–N), an increase in the stretching frequency is observed in complexes compared to the free ligand, with values ranging from 1097 to 1066 cm^−1^ for the complexes and from 1064 cm^−1^ to 1038 cm^−1^ for the ligands, respectively [27,28]. Based on the similarity with other nickel(II) complexes with Schiff bases and pyridine or triphenylphosphine reported in the literature, the bands in the 690–694 cm^−1^ region can be assigned to δ(Py) and ν(Ni–PPh_3_) vibrations [18,30,31].

### 2.4. Electronic Spectra

The electronic spectra of the Schiff bases and their complexes (**1**–**4**) were obtained to investigate the possible electronic transitions at concentrations of 2 × 10^−5^ mol·L^−1^ in methanol and dimethylformamide (Figures S15–S18). The wavelengths representing π–π* transitions of the azomethine group, n–π* transitions, and ligand–metal charge transfer (LMCT) transitions are listed in Table 3.

All compounds exhibited bands in high-energy regions, attributed to the π→π* transition of the azomethine function. In the ligands, these values ranged between 283–284 nm for H_2_L^1^ and 262–294 nm for H_2_L^2^. In the complexes, a decrease in the wavelength of this transition was observed, indicating a hypochromic shift upon complexation, with values between 229–281 nm [40]. For the ligands H_2_L^1^ and H_2_L^2^ in methanol, n→π* transitions were observed at wavelengths of 415 nm and 445 nm, respectively, while in dimethylformamide, these transitions were found at 431 nm and 451 nm. The complexes exhibited broad bands in the range of 441–501 nm, attributed to charge transfer S→Ni(II), as seen in other studies, suggesting the coordination by the thiolate group [17,30,38,41]. Furthermore, the complexes formed with the thiosemicarbazone ligand exhibited several ligand-to-metal charge transfer transitions, whereas the complexes with the dithiocarbazate ligand exhibited fewer such transitions. The d-d transitions for Ni(II) typically occur in the visible region of the spectrum. Still, distinct d-d transitions were not observed in the complexes, presumably due to their low intensity and overlap with the more intense ligand-based and LMCT bands. Despite the high structural similarity, small variations in the ligands can alter the molecular configuration, affecting the optical properties and intermolecular interactions and causing variations in the number of observable peaks in the UV–visible spectrum.

### 2.5. Mass Spectrometry

Electrospray ionization mass spectra in positive mode ESI(+)–MS(/MS) were acquired to investigate the actual species present in solution for H_2_L^1^, H_2_L^2^, and their complexes (**1**–**4**) at a concentration of 50 μM (Figures S19–S28). The ESI(+)–MS/MS spectrum for the ligand H_2_L^1^ peaks at *m*/*z* = 372.0450, corresponding to the [M+H]^+^ ion. In contrast, the base peak at *m*/*z* = 246.0310 was attributed to the loss of the β-diketone moiety containing the thiophene group. Additional fragment ions were identified at *m*/*z* = 219.0198, assigned to the loss of both the thiophene and CF_3_ groups from the β-diketone, along with signals corresponding to the terminal thiosemicarbazone moiety and half of the β-diketone at *m*/*z* = 136.0216 and *m*/*z* = 110.99606, respectively. Similarly, H_2_L^2^ displayed a peak at *m*/*z* = 403.1103, representing the [M+H]^+^ ion, and a base peak at *m*/*z* = 277.0072, corresponding with partial β-diketone loss containing the thiophene group. Fragment peaks at *m*/*z* = 110.9906, corresponding to the half β-diketone, and *m*/*z* = 91.00561, attributed to the loss of the S-benzyl moiety from the dithiocarbazate, were detected.

**Figure 6:**
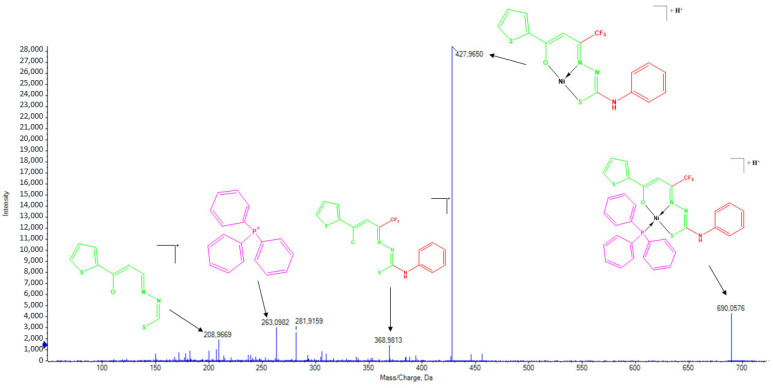
ESI(+)–MS/MS spectrum of the complex (**1**).

Complexes (**1**) and (**2**), due to their structural similarity, exhibited similar fragmentation patterns in their ESI(+)–MS/MS spectra. For (**1**), a peak at *m*/*z* = 690.0576 was observed, corresponding to the [M+H]^+^ ion, with the base peak at *m*/*z* = 427.9650 attributed to the loss of the triphenylphosphine coligand (Figure 6). Complex (**2**) exhibited a peak at *m*/*z* = 507.3280 for the [M+H]^+^ ion and a base peak at *m*/*z* = 427.9645, associated with the loss of the pyridine group. In both spectra, the presence of the deprotonated free ligand was observed at *m*/*z* = 368.9810, along with a fragment corresponding to the ligand without the terminal thiosemicarbazone moiety at *m*/*z* = 208.9669. Additionally, peaks corresponding to triphenylphosphine at *m*/*z* = 263.0982 for complex (**1**) and pyridine at *m*/*z* = 80.0512 for complex (**2**) were observed [17].

**Figure 7:**
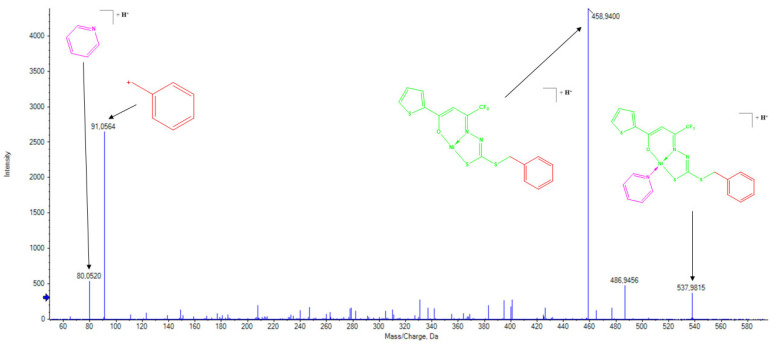
ESI(+)–MS/MS spectrum of the complex (**4**).

Due to their structural similarity, the complexes with dithiocarbazate ligands exhibited analogous fragmentation patterns. In the ESI(+)–MS/MS spectrum of (**3**), a peak at *m*/*z* = 721.0333 was observed for the [M+H]^+^ ion, while the base peak at *m*/*z* = 458.9412 was attributed to the loss of the triphenylphosphine coligand. The ESI(+)–MS/MS of (**4**) displayed a peak at *m*/*z* = 537.9815 for the [M+H]^+^ ion, with the base peak at *m*/*z* = 458.9400 corresponding to the loss of the pyridine group (Figure 7). Both spectra showed a fragment peak at *m*/*z* = 91.0552, assigned to the terminal dithiocarbazate moiety, along with triphenylphosphine at *m*/*z* = 263.0984 for (**3**) and pyridine at *m*/*z* = 80.0520 for (**4**) [17]. The isotopic distribution patterns observed in the ESI(+)–MS spectra strongly support the proposed structures of the synthesized compounds, as the experimental data demonstrated excellent agreement with theoretical values [42].

### 2.6. ^1^H, ^19^F, and ^31^P NMR Spectra

The ^1^H NMR spectra of the synthesized ligands and complexes (**1**–**4**) are presented in Figures S28–S33, while the corresponding chemical shift values and coupling constants (J) are summarized in Table 4 and Table S2–S7. In all compounds, the most shielded signals in the spectra correspond to singlets attributed to CH_2_ groups, with chemical shifts ranging from 4.46 ppm to 4.67 ppm in the ligands and from 4.10 ppm to 4.33 ppm in the complexes (**3**) and (**4**), associated with the terminal dithiocarbazate moiety. Upon complex formation, deprotonation of the C6 carbon occurs, leading to electronic delocalization throughout the structure due to coordination with the metal center [27,41,43]. This delocalization reduces the electron density on the remaining hydrogen atom bound to this carbon, increasing its unshielding effect and shifting the signal to higher chemical shift values [27,41,43]. In complexes (**1**) and (**2**), this shift was observed at 6.26 ppm and 6.17 ppm, respectively, whereas in complexes (**3**) and (**4**), these signals overlapped with the aromatic region. Regarding the most unshielded chemical environments, the hydrogen atoms were attributed to the amino groups in the ligands, with chemical shifts at 11.19 ppm and 13.20 ppm for H_2_L^1^ and H_2_L^2^, respectively [22,28,33]. The remaining signals were attributed to aromatic hydrogen atoms. Although some of these aromatic hydrogens are chemically non-equivalent, their signals appeared in the same regions. The ^19^F NMR spectra of all compounds are presented in Figure S35; the spectra display characteristic chemical shifts for the trifluoromethyl group in the range of −62.04 to −68.40 ppm, consistent with values reported for analogous CF_3_-containing compounds in the literature [44,45]. The ^31^P NMR spectra of the free PPh_3_ and the ligands and complexes (**1**) and (**3**), shown in Figure S36, exhibit a downfield-shifted singlet at 22.92 and 22.30 ppm, respectively, for (**1**) and (**3**), consistent with the presence of a single coordinated triphenylphosphine ligand in the nickel(II) complexes. These chemical shifts align with those reported for analogous Ni(II)-PPh_3_ systems, confirming the expected coordination environment [46,47].

### 2.7. Biological Activity Analysis

**Figure 8:**
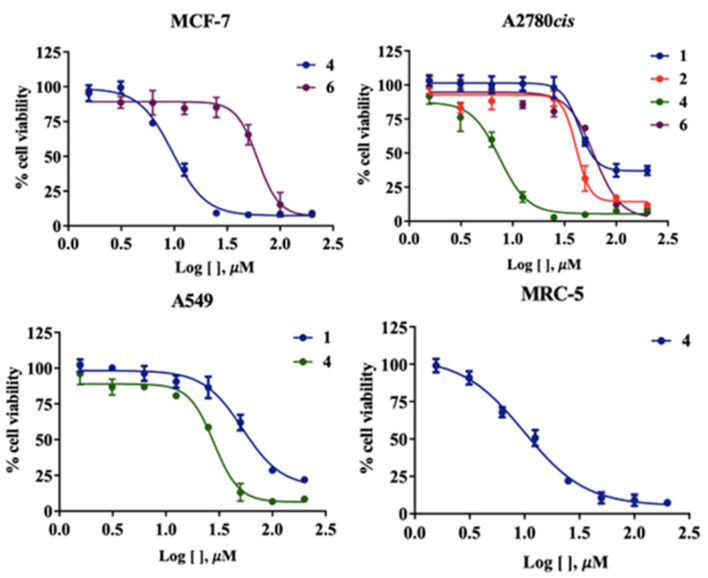
Evaluation of cytotoxic effects by MTT assay in 48 h. DMSO at 0.5% did not affect the cell viability of the cell lines.

DMSO solutions (10^−3^ M) of the complexes (**1**–**4**) were subjected to molar conductance measurements at room temperature over 48 h, and the observed values are presented in Table S8. The complexes showed low molar conductance values, which indicates that they are present in their neutral form. A comparative cytotoxicity assay explored the differences between nickel(II) complexes derived from thiosemicarbazone and dithiocarbazate ligands. Additionally, we investigated the influence of the coligands triphenylphosphine and pyridine in the complexes. The effectiveness of the compounds in inhibiting cell viability was concentration- and time-dependent. The analysis involved varying concentrations between 1.56 and 200 µM over 48 h using an MTT assay, with untreated cells serving as the control. Table 5 presents the IC_50_ values for the free ligands, synthesized complexes, and cisplatin, which served as reference compounds in this study [48]. The compounds’ proliferative ability in 48 h was significantly reduced with increasing concentration (Figure 8).

For the MCF-7 breast cancer cell line, no cytotoxic activity was observed for the free ligands at concentrations below 100 µM. In contrast, only the complexes containing pyridine as a coligand exhibited cytotoxic activity, with IC_50_ values of 8.48 µM for complex (**2**) and 56.54 µM for complex (**4**). In the cisplatin-resistant ovarian cancer cell line A2780cis, both free ligands demonstrated cytotoxic activity with IC_50_ values of 42.78 µM and 78.93 µM for H_2_L^1^ and H_2_L^2^, respectively. Upon complexation, an increase in cytotoxic activity was observed when pyridine was used as the coligand, whereas the use of triphenylphosphine resulted in reduced cytotoxicity. For the A549 lung cancer cell line, cytotoxic activity was observed only for the thiosemicarbazone ligand and complex (**2**) with IC_50_ values of 51.38 µM and 27.18 µM, respectively, indicating enhanced cytotoxicity upon coordination. For the nontumoral lung cell line MRC-5, only complex (**2**) showed an IC_50_ value of 9.89 µM.

The results demonstrate that Ni(II) complexes containing pyridine groups exhibited greater cytotoxic activity than those with triphenylphosphine, similar to related complexes [17,30]. This indicates that bulky groups coordinated to the metal centers may increase lipophilicity by reducing their bioavailability [17,31]. In addition, the cytotoxicity of the synthesized compounds against the A2780cis cell line is particularly noteworthy, as this cell line has demonstrated resistance to cisplatin-based treatments [49]. The synthesized compounds H_2_L^1^, H_2_L^2^, and complexes (**2**) and (**4**) may represent promising candidates for alternative therapies, as they exhibit cytotoxic activity against this cisplatin-resistant cell line while demonstrating lower toxicity to healthy cells, such as MRC-5. Although MRC-5 represents a different tissue type, the results suggest a degree of selectivity of these compounds towards tumor cells, except for complex (**2**), which was the only compound with an IC_50_ value below 100 µM. While complex (**2**) demonstrates prominent cytotoxicity against MCF-7 and A2780cis cell lines, its comparable toxicity toward nontumoral MRC-5 lung cells indicates limited therapeutic selectivity. In contrast, complex 4 shows moderate tumor cell inhibition with no observable toxicity to MRC-5 cells at ≤100 μM, suggesting a potentially safer pharmacological profile despite reduced potency. This contrast highlights the balance between efficacy and selectivity in drug development.

## 3. Materials and Methods

### 3.1. Materials, Methods, and Instruments

The reagents and solvents were obtained from Merck-Brazil and used without further purification. The complexation studies were performed using fresh pyridine. The FT–IR spectra of the compounds were obtained from KBr pellets using an FT–IR Varian 640 spectrometer (Agilent Technologies, Santa Clara, CA, USA) in the range of 4000–400 cm^−1^, with 32 scans at a resolution of 4 cm^−1^. Electrospray ionization–mass spectrometry analysis (ESI–MS) was performed on an AB Sciex Triple TOF 5600+ mass spectrometer (SCIEX, Marlborough, MA, USA) in positive mode, with a voltage of 5500 V and source temperature of 200 °C. ^1^H, ^19^F, and ^31^P nuclear magnetic resonance spectra were collected on a Bruker Ascend 600 MHz (UC Davis RMN Facility, Davis, CA, USA), with TMS as internal reference and DMSO–*d_6_* as solvent for ^1^H experiments and PF_6_ as intermetal reference and D_2_O as solvent for ^19^F and ^31^P experiments. Elemental analysis of CHN was performed with a Perkin Elmer/Series II 2400 analyzer (Perkin Elmer, Shelton, CT, USA). The reported elemental analysis values are from a single measurement, even though the analysis was performed in triplicate. Electronic spectra were obtained using a UV–Vis–NIR Varian Cary 5000 spectrophotometer (Agilent Technologies, Santa Clara, CA, USA) using 20 μM solutions prepared in methanol and dimethylformamide. Complexes (**1**–**4**) were synthesized based on procedures previously described for similar compounds [17,18,30].

### 3.2. Synthesis of Thiosemicarbazone Ligand (H_2_L^1^)

H_2_L^1^ was synthesized based on a patterning procedure [50]. An amount of 666.57 mg (3 mmol) of thenoyltrifluoroacetone was added to an ethanolic solution (40 mL) of 501.69 mg (3 mmol) of 4–phenyl–thiosemicarbazide and was stirred under reflux for 4 h with a few drops of H_2_SO_4_. Yellow crystals suitable for X-ray diffraction were obtained and filtered after slow evaporation of the solvent of the mother solution. Yield: 1017 mg (91.4%). Melting point: 186–187 °C. Elemental analysis calculated for C_15_H_12_F_3_N_3_OS_2_ (%): C. 48.51; H. 3.26; N. 11.31. Found (%): C. 48.82; H. 3.69; N. 11.56. IR bands (KBr, ν/cm^−1^): ν(N–H) 3333 and 3304; ν(C=N) 1597; ν(C=S) 1307; ν(N–N) 1038; ν(C–S) 728. ^1^H NMR (DMSO–d_6_, ppm): 11.72 (s, 1H, N–H), 10.19 (s, 1H, N–H), 8.12–8.10 (m, 1H, –CH), 8.10–8.08 (m, 1H, –CH), 7.53 (d, 1H, Ar), 7.39 (dd, 1H, Ar), 7.33 (dd, 1H, Ar), 7.25 (t, 1H, Ar), 4.67 (s, 2H, CH_2_). ^19^F NMR (D_2_O, ppm): −67.57 (s, CF_3_). ^31^P NMR (D_2_O, ppm): −6.50 (s, PPh_3_-free). UV–vis (MeOH): λmax = 284 nm and 415 nm. UV–vis (DMF): λmax = 283 nm and 431 nm. ESI–MS [M+H]^+^ (calcd, found, *m*/*z*) = 372.0447/372.0449.

### 3.3. Synthesis of Dithiocarbazate Ligand (H_2_L^2^)

H_2_L^2^ was obtained following the procedures in the related literature [33,51]. A mixture of 0.194 mL (1 mmol) of hydrazine hydrate and 56.1 mg (1 mmol) of KOH in 15 mL of ethanol was cooled to 5 °C and refluxed for 1 h. In this solution, 0.06 mL (1 mmol) of carbon disulfide is used. The mixture was kept in an ice bath, and 0.12 mL of benzyl bromide was added dropwise with vigorous stirring. After 1 h, 222.19 mg of thenoyltrifluoroacetone was added, and the final mixture was refluxed and heated for 1 h. Yellow crystals were obtained after some days with the evaporation of the mother solution at room temperature. Yield: 56.2 mg (14%). Melting point: 101 °C. Elemental analysis calculated for C_16_H_13_F_3_N_2_OS_3_ (%): C. 47.75; H. 3.26; N. 6.96. Found (%): C. 47.43; H. 3.26; N. 7.27. IR bands (KBr, ν/cm^−1^): ν(N–H) 3108; ν(C=N) 1594; ν(C=S) 1306; ν(N–N) 1064; ν(C–S) 732. ^1^H NMR (DMSO–d_6_, ppm): 13.20 (s, 1H, N–H), 8.10 (d, 1H, –CH), 8.07 (d, 1H, –CH), 7.42 (d, 1H, Ar), 7.36–7.27 (m, 4H, Ar), 4.65 (s, 2H, –CH_2_), 4.46 (s, 2H, CH_2_). ^19^F NMR (D_2_O, ppm): −68.40 (s, CF_3_). ^31^P NMR (D_2_O, ppm): −6.50 (s, PPh_3_-free). UV–vis (MeOH): λmax = 262 nm, 295 nm, and 445 nm. UV–vis (DMF): λmax = 294 nm and 451 nm. ESI–MS [M+H]^+^ (calcd, found, *m*/*z*) = 403.0215/403.0213.

### 3.4. Synthesis of [Ni(L^1^)(PPh_3_)] (***1***)

Initially, 52 mg (0.2 mmol) of triphenylphosphine was dissolved in 5 mL of methanol and added to a solution of 23.7 mg (0.1 mmol) of NiCl_2_·6H_2_O in 5 mL of methanol, and the mixture was refluxed for 10 min. Subsequently, a solution of H_2_L^1^ (37.1 mg, 0.1 mmol) in 5 mL of methanol was added, and the reaction was continued under reflux for 2 h. Red crystals appropriate for single-crystal X-ray diffraction analysis were filtered off from the mother solution. Yield: 47.1 mg (68.3%). Melting point: 204–205 °C. Elemental analysis calculated for C_33_H_25_F_3_N_3_OS_2_PNi (%): C. 57.41; H. 3.65; N. 6.09. Found (%): C. 57.42; H. 3.34; N. 6.07. IR bands (KBr, ν/cm^−1^): ν(C=N) 1544; ν(C=C) 1506 and 1434; ν(N–N) 1097; ν(C–S) 746; ν(PPh_3_) 692. ^1^H NMR (DMSO–d_6_, ppm): 9.40 (s, 1H, N–H), 7.78 (t, 6H, PPh_3_), 7.64–7.51 (m, 13H, Ar and PPh_3_), 7.20 (t, 2H, Ar), 6.92–6.89 (m, 1H, –CH), 6.87 (t, 1H, Ar), 6.26 (s, 1H, –CH). ^19^F NMR (D_2_O, ppm): −63.63 (s, CF_3_). ^31^P NMR (D_2_O, ppm): 22.92 (s, PPh_3_). UV–vis (MeOH): λmax = 229 nm, 273 nm, 361 nm, 464 nm, and 494 nm. UV–vis (DMF): λmax = 445 nm, 469 nm, and 500 nm. ESI–MS [M+H]^+^ (calcd, found, *m*/*z*) = 690.0555/690.0563.

### 3.5. Synthesis of [Ni(L^1^)(Py)] (***2***)

The complex was synthesized by reaction of pyridine (16 μL, 0.2 mmol) in ethanol (5 mL) with Ni(Ac)_2_·4H_2_O (24.8 mg, 0.1 mmol) in ethanol (5 mL). The mixture was refluxed for 10 min, followed by the addition of H_2_L^1^ (37.1 mg, 0.1 mmol) in ethanol (5 mL). The resulting solution was then refluxed for 2 h. Red crystals appropriate for single-crystal X-ray diffraction analysis were obtained in the refrigerator and filtered from the mother solution. Yield: 34.3 mg (67.8%). Melting point: 149–150 °C. Elemental analysis calculated for C_20_H_15_F_3_N_4_OS_2_Ni (%): C. 47.36; H. 2.98; N. 11.05. Found (%): C. 47.20; H. 2.68; N. 10.83. IR bands (KBr, ν/cm^−1^): ν(C=N) 1544; ν(C=C) 1510 and 1413; ν(N–N) 1066; ν(C–S) 746; δ(Py) 694. ^1^H NMR (DMSO–d_6_, ppm): 9.40 (s, 1H, N–H), 9.06 (s, 2H, –CH Py), 8.04 (s, 1H, –CH Py), 7.72–7.51 (m, 6H, Ar and Py), 7.21 (t, 2H, Ar), 7.14–7.07 (m, 1H, –CH), 6.88 (t, 1H, Ar), 6.17 (s, 1H, –CH). ^19^F NMR (D_2_O, ppm): −63.13 (s, CF_3_). UV–vis (MeOH): λmax = 257 nm, 333 nm, 441 nm, 444 nm, and 499 nm. UV–vis (DMF): λmax = 281, nm, 444 nm, 469 nm, and 501 nm. ESI–MS [M+H]^+^ (calcd, found, *m*/*z*) = 507.0065/507.0064.

### 3.6. Synthesis of [Ni(L^2^)(PPh_3_)] (***3***)

Triphenylphosphine (52 mg, 0.2 mmol) was dissolved in methanol (5 mL) and added to a solution of NiCl_2_·6H_2_O (23.7 mg, 0.1 mmol) in methanol (5 mL). The mixture was refluxed for 10 min. Subsequently, H_2_L^2^ (40.4 mg, 0.1 mmol) in methanol (5 mL) was added directly to the reaction mixture, and reflux was continued for 2 h. Red crystals appropriate for single-crystal X-ray diffraction analysis were obtained in the refrigerator and filtered off from the mother solution. Yield: 54.1 mg (75.1%). Melting point: 182–183 °C. Elemental analysis calculated for C_34_H_26_F_3_N_2_OPS_3_Ni (%): C. 56.61; H. 3.63; N. 3.88. Found (%): C. 56.88; H. 3.59; N. 4.02. IR bands (KBr, ν/cm^−1^): ν(C=N) 1527; ν(C=C) 1400; ν(N–N) 1095; ν(CSS) 991; ν(C–S) 746; ν(PPh_3_) 692. ^1^H NMR (DMSO–d_6_, ppm): 7.80–7.65 (m, 6H, PPh_3_), 7.65–7.42 (m, 13H, Ar and PPh_3_), 7.34–7.29 (m, 4H, Ar), 7.29–7.22 (m, 1H, Ar), 4.33 (s, 2H, –CH_2_). ^19^F NMR (D_2_O, ppm): −62.04 (s, CF_3_). ^31^P NMR (D_2_O, ppm): 22.30 (s, PPh_3_). UV–vis (MeOH): λmax = 251 nm, 275 nm, and 449 nm. UV–vis (DMF): λmax = 445 nm. ESI–MS [M+H]^+^ (calcd, found, *m*/*z*) = 721,0323/721,0316.

### 3.7. Synthesis of [Ni(L^2^)(Py)] (***4***)

Complex **4** was synthesized by reacting pyridine (16 μL, 0.2 mmol) in methanol (5 mL) with NiCl_2_·6H_2_O (23.7 mg, 0.1 mmol) in methanol (5 mL). The mixture was refluxed for 10 min, followed by the addition of H_2_L^2^ (40.4 mg, 0.1 mmol) in methanol (5 mL). The resulting solution was then refluxed for 2 h. Red crystals appropriate for single-crystal X-ray diffraction analysis were filtered from the mother solution. Yield: 37.9 mg (70.7%). Melting point: 152–154 °C. Elemental analysis calculated for C_21_H_15_F_3_N_3_OS_3_Ni (%): C. 46.95; H. 2.81; N. 7.82. Found (%): C. 46.51; H. 3.02; N. 7.73. IR bands (KBr, ν/cm^−1^): ν(C=N) 1529; ν(C=C) 1405; ν(CSS) 991; ν(C–S) 759; δ(Py) 694. ^1^H NMR (DMSO–d_6_, ppm): 10.39 (s, 2H, Py), 8.16–7.79 (m, 13H, Ar and Py), 7.46–7.37 (m, 1H, Ar), 7.37–7.26 (m, 4H, Ar), 7.26–7.15 (m, 1H, Ar), 4.10 (s, 2H, –CH_2_). ^19^F NMR (D_2_O, ppm): −62.33 (s, CF_3_). UV–vis (MeOH): λmax = 257 nm and 454 nm. UV–vis (DMF): λmax = 455 nm. ESI–MS [M+H]^+^ (calcd, found, *m*/*z*) = 537.9833/537.9813.

### 3.8. Crystal Structure Determination

The X-ray data collection was performed on a Bruker CCD SMART APEX II single-crystal diffractometer with Mo Kα radiation (0.71073 Å) at room temperature (296 K). The collected data were processed with SAINT [52], and SADABS [53] was used for absorption correction. The structures were solved with direct methods using the SHELXS program [54], and the positions of the nonhydrogen atoms were determined with subsequent Fourier difference map analyses. The refinement was accomplished with SHELXL–2018 using least squares minimization [55]. The Olex2 program [56] was used for the solution and refinement of the structures. Molecular graphics were generated via MERCURY 2024.2.0 software [57]. Table S9 summarizes the crystal data, experimental details, and refinement results.

### 3.9. Hirshfeld Surface

The Hirshfeld surfaces (HS) and the 2D fingerprint plots (FP) of the complexes (**1**–**4**) were generated using the software CrystalExplorer 21.5 [35]. The crystallographic information files (CIFs) obtained from the single-crystal X-ray diffraction analysis were used as input files. The normalized contact distance (d_norm_) is defined in terms of functions of distance *d_i_* and *d_e_* and the van der Waals radii of the atoms. Once the surface has been calculated and the distance between the nearest nucleus inside the surface (*d_i_*) and the nearest nucleus outside the surface (*d_e_*) is lower, closer, or bigger than the sum of the van der Waals radii of them, the surface will indicate the presence or absence of contacts. Two-dimensional fingerprint plots were obtained with the combination of *d_i_* and *d_e_* distances, on the scale of 0.4 to 2.6 Å, to summarize the contacts present in the crystal structure of the complexes.

### 3.10. Anticancer Activity

#### 3.10.1. Cell Culture

The cytotoxic activity of the synthesized compounds was evaluated against human cell lines, including breast cancer (MCF–7), cisplatin-resistant ovarian cancer (A2780cis), lung cancer (A549), and nontumor lung cells (MRC–5). The A549 and MRC-5 cell lines were cultured in Dulbecco’s Modified Eagle’s Medium (DMEM), while the MCF-7 and A2780cis cell lines were cultured in Roswell Park Memorial Institute (RPMI–1640). Both cultures were supplemented with 10% fetal bovine serum (FBS), penicillin (100 IU/mL), streptomycin (100 mg/mL), and L-glutamine (2 mM). The cells were grown as monolayers in disposable 25 cm^2^ flasks and incubated at 37 °C in a humidified atmosphere containing 5% CO_2_.

#### 3.10.2. Cell Viability Analysis

The cytotoxicity activity of the compounds was assessed using a colorimetric MTT (3-(4,5-dimethylthiazol-2-yl)-2,5-diphenyltetrazolium bromide) assay [48]. The cells were trypsinized, counted, and adjusted to the appropriate concentration and were seeded into 96-well plates at a density of 1.5 × 10^4^ cells/well. The plates were incubated at 37 °C in a 5% CO_2_ atmosphere for 24 h to allow cell adhesion. Subsequently, the compounds, solubilized in dimethyl sulfoxide (DMSO) at varying concentrations (200—1.56 µM), were added to the wells, and the plates were incubated for 48 h. Cells treated with 0.5% DMSO served as the control. After the 48-h treatment period, 50 µL of MTT solution (0.6 mg/mL in phosphate-buffered saline, PBS) was added to each well, followed by incubation for 4 h. The formazan crystals formed from the wells were separated from the solution and then solubilized by the addition of 100 µL of DMSO per well. The absorbance was measured at 570 nm using a Varioskan LUX Multimode Microplate Reader (VL000D0, Waltham, MA 02451, USA). All experiments were performed in triplicate. The half-maximal inhibitory concentration (IC_50_) values were determined from dose–response curves using GraphPad Prism version 8 software [48].

## 4. Conclusions

Four new Ni(II) complexes with thiosemicarbazone and dithiocarbazate were successfully synthesized. The crystal structures were elucidated through single-crystal X-ray diffraction, which was consistent with the data obtained from spectroscopic and spectrometric analyses. All complexes exhibited a square planar geometry, with the ligands displaying double deprotonation and coordinated by tridentate mode through an *ONS* donor system. The coordination sphere was completed by a triphenylphosphine or pyridine coligand. Hirshfeld surface analysis was employed to estimate the topography of intermolecular interactions, while fingerprint plots provided quantitative insights into the most significant contributions to the crystal packing, primarily attributed to H⋯H and C⋯H interactions across all complexes. Biological assays show interesting results, particularly for the compounds containing pyridine as a substituent in their coordination sphere. Complex (**2**) exhibits potent cytotoxicity against breast and ovarian cancer cell lines compared to cisplatin; however, its lack of selectivity for nontumor cells raises concerns about its therapeutic potential. In contrast, complex (**4**) demonstrates a more promising profile, with moderate tumor cell inhibition and no observable toxicity to normal cells at concentrations up to 100 μM, highlighting the importance of balancing efficacy and selectivity in the development of cancer treatments.

## Figures and Tables

**Table 1:** Selected bond lengths (Å) and bond angles (°) for the complexes (**1**–**4**).

Bond Lengths (Å)				
	(1)	(2)	(3)	(4)
C5–O1	1.295(4)	1.280(11)	1.297(4)	1.299(7)
C7–N1	1.324(4)	1.312(10)	1.321(5)	1.325(7)
N1–N2	1.392(3)	1.408(10)	1.396(4)	1.406(5)
N2–C9	1.293(4)	1.309(11)	1.291(5)	1.292(7)
C9–S2	1.732(3)	1.745(9)	1.732(4)	1.735(6)
Ni1–O1	1.841(2)	1.857(6)	1.849(3)	1.835(4)
Ni1–N1	1.898(3)	1.863(7)	1.874(3)	1.859(4)
Ni1–S2	2.112(10)	2.143(3)	2.137(12)	2.128(16)
Ni1–PPh_3_	2.204(10)	–	2.210(12)	–
Ni–Py	–	1.919(8)	–	1.903(5)
**Bond Angles (°)**				
	**(1)**	**(2)**	**(3)**	**(4)**
N1–Ni1–S2	87.28(8)	86.7(2)	87.46(10)	88.04(14)
O1–Ni1–N1	95.98(10)	95.6(3)	93.99(13)	95.82(19)
O1–Ni–S2	175.94(8)	177.6(19)	174.23(10)	175.39(14)
O1–Ni1–PPh_3_	85.87(7)	–	86.50(9)	–
S2–Ni1–PPh_3_	90.85(4)	–	92.24(5)	–
N1–Ni1–PPh_3_	178.12(8)	–	178.07(10)	–
O1–Ni1–Py	–	86.7(3)	–	86.11(18)
S2–Ni1–Py	–	91.0(2)	–	90.00(13)
N1–Ni1–Py	–	174.6(3)	–	177.96(19)

**Table 2:** Angular strain and strain (cm^−1^) frequencies of the normal vibration modes selected for H_2_L^1^, H_2_L^2^, and the complexes (**1**–**4**).

	H_2_L^1^	H_2_L^2^	(1)	(2)	(3)	(4)
ν(C=S)	1307	1306	–	–	–	–
ν(C–S)	728	732	746	746	746	759
ν(N–N)	1038	1064	1097	1066	1095	–
ν(C=O)	1651	1637	–	–	–	–
ν(C=N)	1597	–	1544	1544	1544	1544
ν(N–H)	3333/3304	3195	3401	3343	–	–
δ(Py)	–	–	–	690	–	694
ν(Ni–PPh_3_)	–	–	692	–	692	–

**Table 3:** Absorption values of the ligands and complexes (**1**–**4**).

		π–π*	Log ε	n–π*	Log ε	LMCT	Log ε
H_2_L^1^	MeOH	284	4.09	415	3.59	–	–
	DMF	283	4.26	431	–	–	–
H_2_L^2^	MeOH	262 and 295	4.03 and 4.10	445	–	–	–
	DMF	294	4.19	451	–	–	–
(**1**)	MeOH	230 and 273	4.13 and 4.14	441	3.88	461 and 490	3.87 and 3.79
	DMF	–	–	445	4.36	465 and 498	4.34 and 4.25
(**2**)	MeOH	257	4.24	336	3.89	462 and 493	3.87 and 3.70
	DMF	279	4.24	446	4.15	468 and 497	4.13 and 4.04
(**3**)	MeOH	254 and 275	4.18 and 4.16	–	–	449	4.08
	DMF	–	–	455	4.29	488	4.10
(**4**)	MeOH	257	4.18	301	3.97	454	4.10
	DMF	–	–	455	4.35	488	4.16

**Table 4:** ^1^H, ^19^F, and ^31^P NMR spectral data of the ligands H_2_L^1^ and H_2_L^2^ and the complexes (**1**–**4**).

Compound	^1^H (ppm)	^19^F (ppm)	^31^P (ppm)
H_2_L^1^	11.72 (s, 1H, N–H), 10.19 (s, 1H, N–H), 8.12–8.10 (m, 1H, –CH), 8.10–8.08 (m, 1H, –CH), 7.53 (d, J = 7.20 Hz, 1H, Ar), 7.39 (dd, J = 7.40 and 7.20 Hz, 1H, Ar), 7.33 (dd, J = 3.89 and 3.74 Hz, 1H, Ar), 7.25 (t, J = 7.40 Hz, 1H, Ar), 4.67 (s, 2H, CH_2_)	−67.57 (s, CF_3_)	−6.50 (s, PPh_3_-free)
H_2_L^2^	13.20 (s, 1H, N–H), 8.10 (m, J = 4.92 Hz, 1H, –CH), 8.07 (m, J = 3.80 Hz, 1H, –CH), 7.42 (d, J = 7.50 Hz, 1H, Ar), 7.36–7.27 (m, 4H, Ar), 4.65 (s, 2H, –CH_2_), 4.46 (s, 2H, CH_2_)	−68.40 (s, CF_3_)	−6.50 (s, PPh_3_-free)
(**1**)	9.40 (s, 1H, N–H), 7.78 (t, 6H, PPh3), 7.64–7.51 (m, 13H, Ar and PPh3), 7.20 (t, 2H, Ar), 6.92–6.89 (m, 1H, –CH), 6.87 (t, J = 7.35 Hz, 1H, Ar), 6.26 (s, 1H, –CH)	−63.63 (s, CF_3_)	22.92 (s, PPh_3_)
(**2**)	9.40 (s, 1H, N–H), 9.06 (s, 2H, –CH Py), 8.04 (s, 1H, –CH Py), 7.72–7.51 (m, 6H, Ar and Py), 7.21 (t, J = 7.31 Hz, 2H, Ar), 7.14–7.07 (m, 1H, –CH), 6.88 (t, J = 7.31 Hz, 1H, Ar), 6.17 (s, 1H, –CH)	−63.13 (s, CF_3_)	–
(**3**)	7.80–7.65 (m, 6H, PPh3), 7.65–7.42 (m, 13H, Ar and PPh3), 7.34–7.29 (m, 4H, Ar), 7.29–7.22 (m, 1H, Ar), 4.33 (s, 2H, –CH_2_)	−62.04 (s, CF_3_)	22.30 (s, PPh_3_)
(**4**)	10.39 (s, 2H, Py), 8.16–7.79 (m, 13H, Ar and Py), 7.46–7.37 (m, 1H, Ar), 7.37–7.26 (m, 4H, Ar), 7.26–7.15 (m, 1H, Ar), 4.10 (s, 2H, –CH_2_)	−62.33 (s, CF_3_)	–

**Table 5:** Cytotoxic activity of H_2_L^1^, H_2_L^2^, and the complexes (**1**–**4**). Results are presented as IC_50_ values (µM) ± SD in 48 h (values estimated by non-linear regression of data from viability assessment).

Compounds	IC_50_ (µM)			
	MCF-7	A2780cis	A549	MRC-5
H_2_L^1^	>100	(42.78 ± 0.66)	(51.38 ± 4.37)	>100
H_2_L^2^	>100	(78.93 ± 1.96)	>100	>100
(**1**)	>100	>100	>100	>100
(**2**)	(8.48 ± 1.37)	(7.20 ± 0.73)	(27.18 ± 2.91)	(9.89 ± 0.17)
(**3**)	>100	>100	>100	>100
(**4**)	(56.54 ± 1.52)	(65.92 ± 3.52)	>100	>100
Cisplatin	(13.98 ± 2.02)	(37.03 ± 5.11)	(11.54 ± 1.19)	(29.09 ± 0.78)

## Data Availability

The datasets generated for this study can be found in the Supplementary Materials.

## Supplementary Materials

The following supporting information can be downloaded at: https://www.mdpi.com/article/10.3390/molecules30173516/s1. Supplementary tables, figures, NMR, UV-vis, ESI(+)-MS, and IR spectra are as detailed in the text (PDF). Crystallographic data are in CIF files.
