# Supplementary material for: Comparative Study of Ni(II) Complexes with Dithiocarbazate- and Thiosemicarbazone-Based Ligands: Synthesis, Crystal Structures, and Anticancer Activity
Source: Molecules. 2025 Aug 28;30(17):3516. doi: 10.3390/molecules30173516 (PMC12430362; doi:10.3390/molecules30173516)
Supplement: Supplementary file 1 [file molecules-30-03516-s001.zip › molecules-3822490(SI).doc.pdf]

Comparative Study of Ni(II) Complexes with Dithiocarbazate and Thiosemicarbazone-based Ligands: Synthesis, Crystal Structures and Anticancer Activity

Supporting Information

Gabriel S. Pessoa[1], Mariana P. Viana[1], Katia M. Oliveira[1] and Claudia C. Gatto [1,*]

[1] Laboratory of Inorganic Synthesis and Crystallography, Institute of Chemistry, University of Brasilia, Brasília 70904-970, DF, Brazil;

*Corresponding author: Claudia C. Gatto (e-mail address: ccgatto@unb.br; ORCID: https://orcid.org/0000-0002-3736-6861) – University of Brasilia (IQ-UnB), LASIC - Laboratory of Inorganic Synthesis and Crystallography, CEP 70904-970, Brasília-DF, Brazil.

Summary

Figure S1. Non-covalent interactions in the crystal arrangement of the complex (1). IV

Figure S2. Non-covalent interactions in the crystal arrangement of the complex (2). IV

Figure S3. Non-covalent interactions in the crystal arrangement of the complex (3). V

Figure S4. Non-covalent interactions in the crystal arrangement of the complex (4). V

Figure S5. Fingerprint plots for (1). VI

Figure S6. Fingerprint plots for (2). VI

Figure S7. Fingerprint plots for (3). VII

Figure S8. Fingerprint plots for (4). VII

Figure S9. FT-IR spectra of H_2L^1. VIII

Figure S10. FT-IR spectra of H_2L^2. VIII

Figure S11. FT-IR spectra of (1). IX

Figure S12. FT-IR spectra of (2). IX

Figure S13. FT-IR spectra of (3). X

Figure S14. FT-IR spectra of (4). X

Figure S15. UV-vis spectra of compounds H_2L^1, (1) and (2) in MeOH. XI

Figure S16. UV-vis spectra of compounds H_2L^1, (1) and (2) in DMF. XI

Figure S17. UV-vis spectra of compounds H_2L^2, (3) and (4) in MeOH. XII

Figure S18. UV-vis spectra of compounds H_2L^2, (3) and (4) in DMF. XII

Figure S19. ESI(+)-MS spectrum of H_2L^1. XIII

Figure S20. ESI(+)-MS spectrum of H_2L^2. XIII

Figure S21. ESI(+)-MS spectrum of (1). XIV

Figure S22. ESI(+)-MS spectrum of (2). XIV

Figure S23. ESI(+)-MS spectrum of (3). XV

Figure S24. ESI(+)-MS spectrum of (4). XV

Figure S25. ESI(+)-MS/MS spectrum of H_2L^1. XVI

Figure S26. ESI(+)-MS/MS spectrum of H_2L^2. XVI

Figure S27. ESI(+)-MS/MS spectrum of (2). XVII

Figure S28. ESI(+)-MS/MS spectrum of (3). XVII

Figure S29. ^{1}H-NMR spectra of H_2L^1. XVIII

Figure S30. ^{1}H-NMR spectra of H_2L^2. XVIII

Figure S31. ^{1}H-NMR spectra of (1). XIX

Figure S32. ^{1}H-NMR spectra of (2). XIX

Figure S33. ^{1}H-NMR spectra of (3). XX

Figure S34. ^{1}H-NMR spectra of (4). XX

Figure S35. ^{19}F-NMR spectra of ligands H_2L^1 and H_2L^2 and complexes (1–4). XXI
Figure S36. ^{31}P-NMR spectra of free PPh_3 in ligands H_2L^1 and H_2L^2 and complexes (1) and (3). XXI
Table S1. Interactions that contribute to the stabilization of the crystal arrangement of complexes (1-4). XXII
Table S2. ^1H-NMR spectrum data for H_2L^1. XXII
Table S3. ^1H-NMR spectrum data for H_2L^2. XXIII
Table S4. ^1H-NMR spectrum data for (1). XXIII
Table S5. ^1H-NMR spectrum data for (2). XXIII
Table S6. ^1H-NMR spectrum data for (3). XXIV
Table S7. ^1H-NMR spectrum data for (4). XXIV
Table S8. Molar conductivity data ($\Omega^{-1}.cm^2.mol^{-1}$) at 0, 24 and 48 hours of DMSO and complexes (1–4). XXIV
Table S9. X-ray diffraction data collection and refinement parameters for the complexes (1-4). XXV

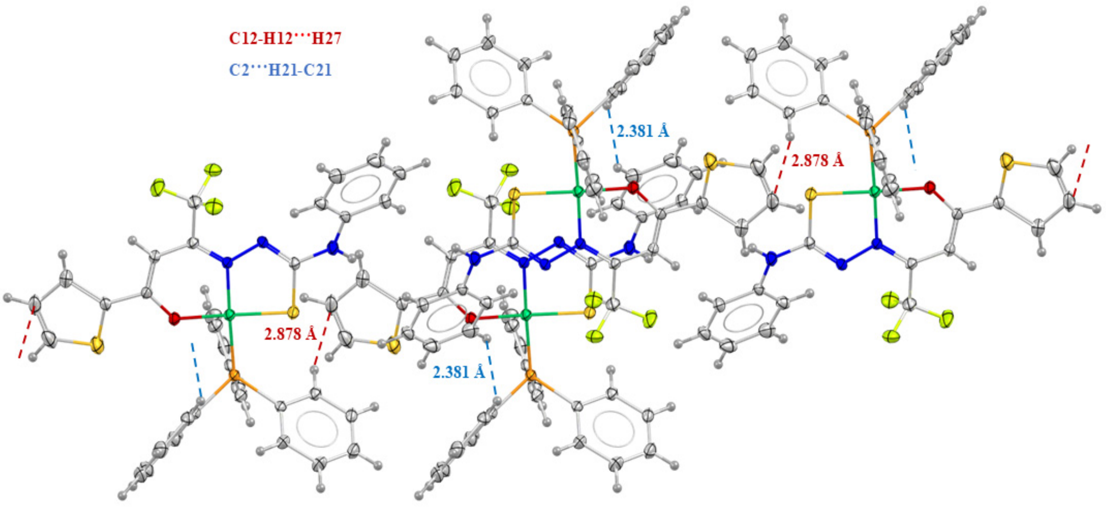

Figure S1. Non-covalent interactions in the crystal arrangement of the complex **(1).**

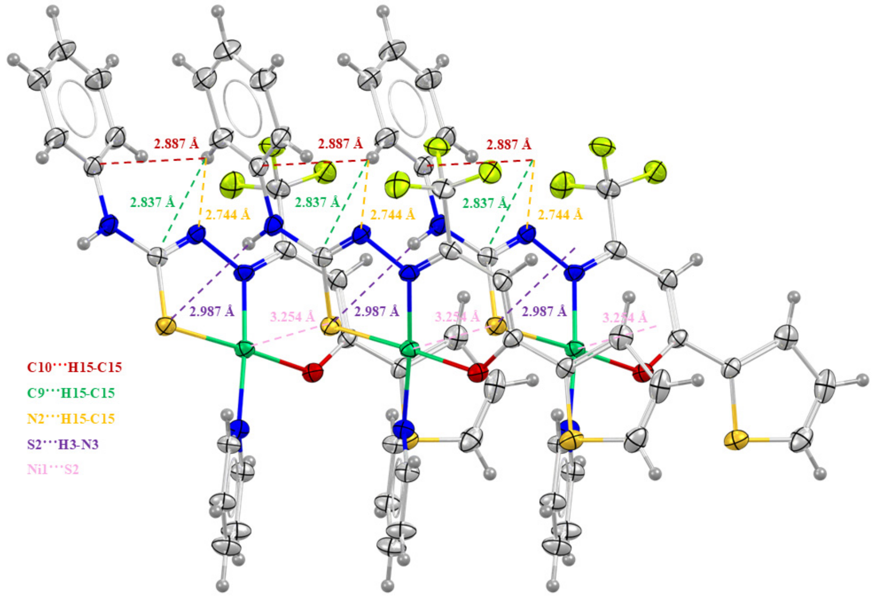

Figure S2. Non-covalent interactions in the crystal arrangement of the complex **(2).**

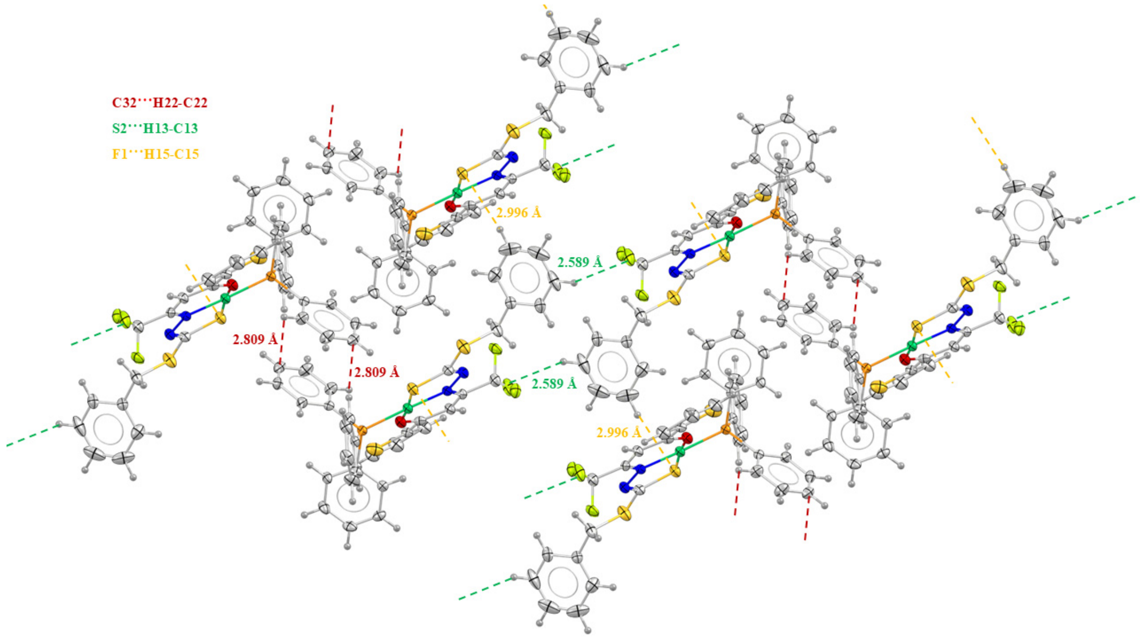

Figure S3. Non-covalent interactions in the crystal arrangement of the complex **(3).**

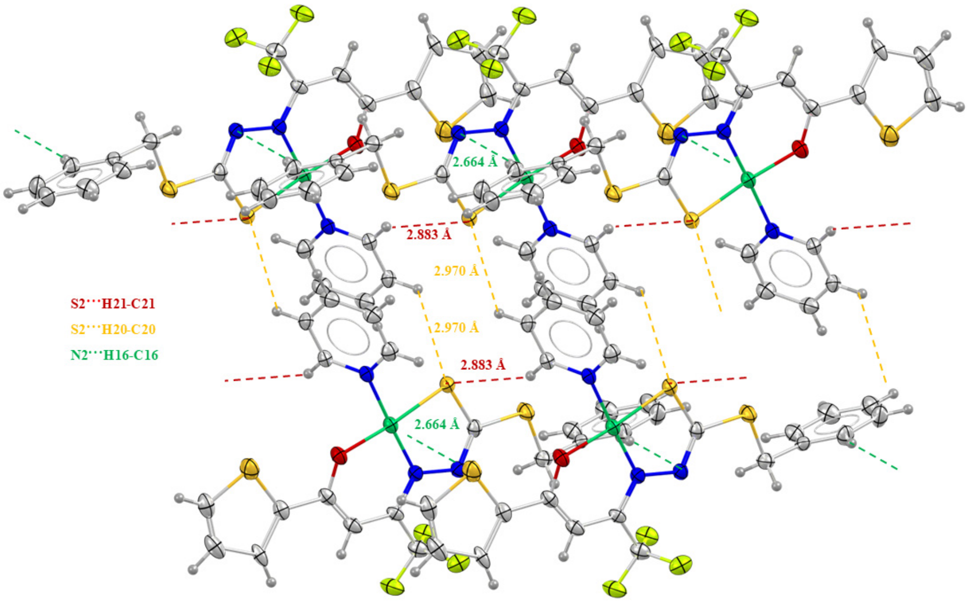

Figure S4. Non-covalent interactions in the crystal arrangement of the complex **(4).**

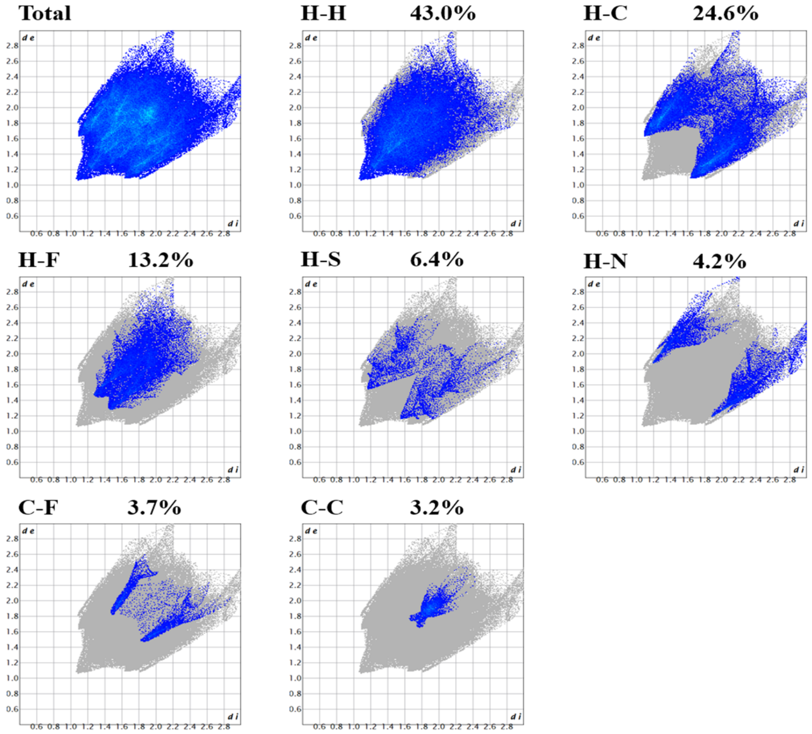

Figure S5. Fingerprint plots for (**1**).

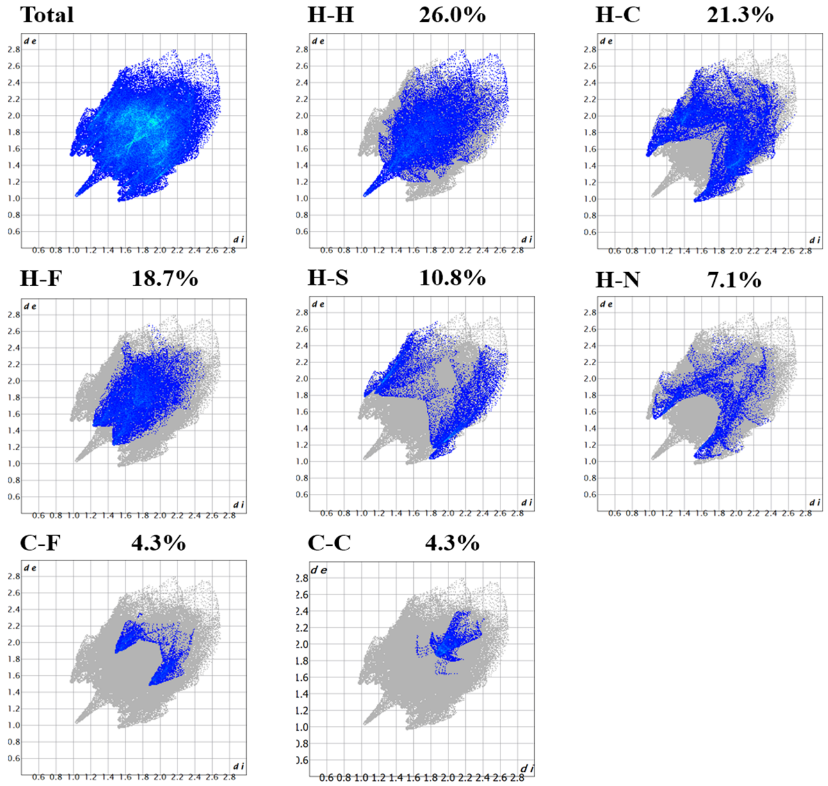

Figure S6. Fingerprint plots for (**2**).

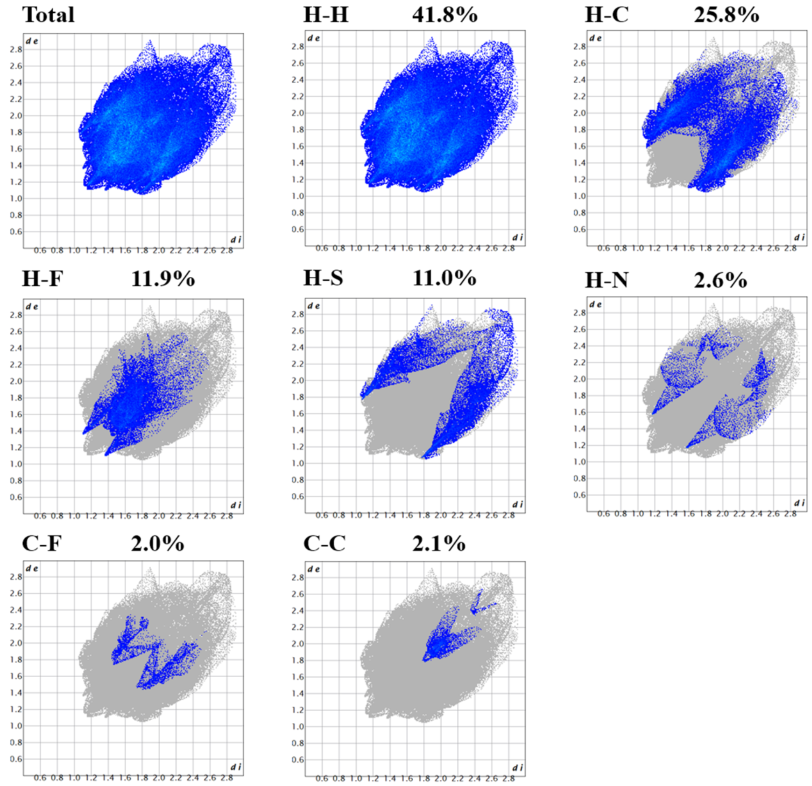

Figure S7. Fingerprint plots for (**3**).

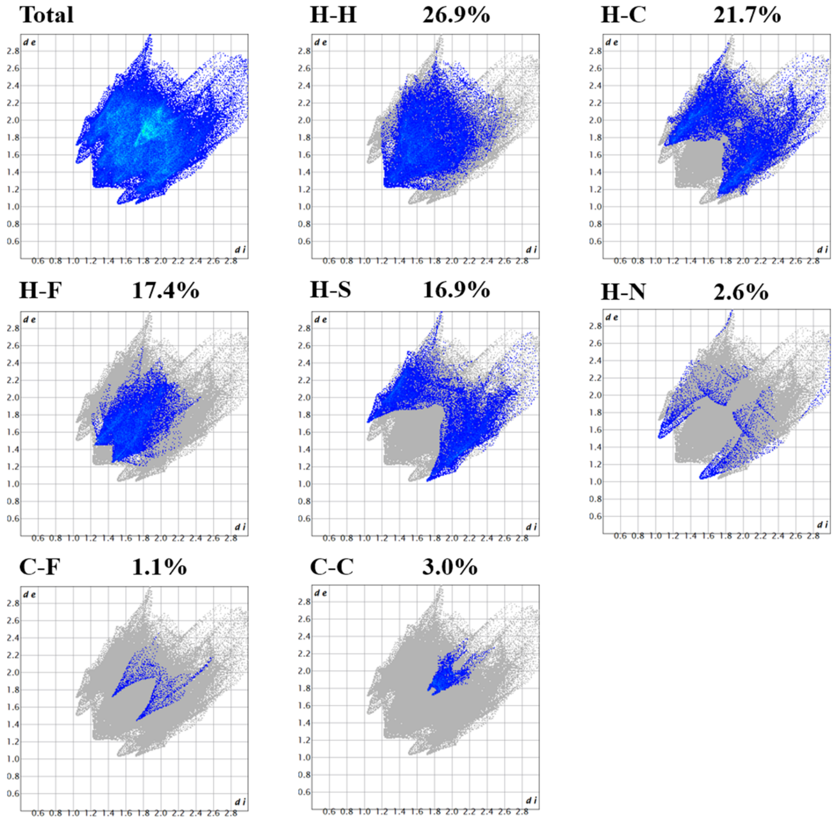

Figure S8. Fingerprint plots for (**4**).

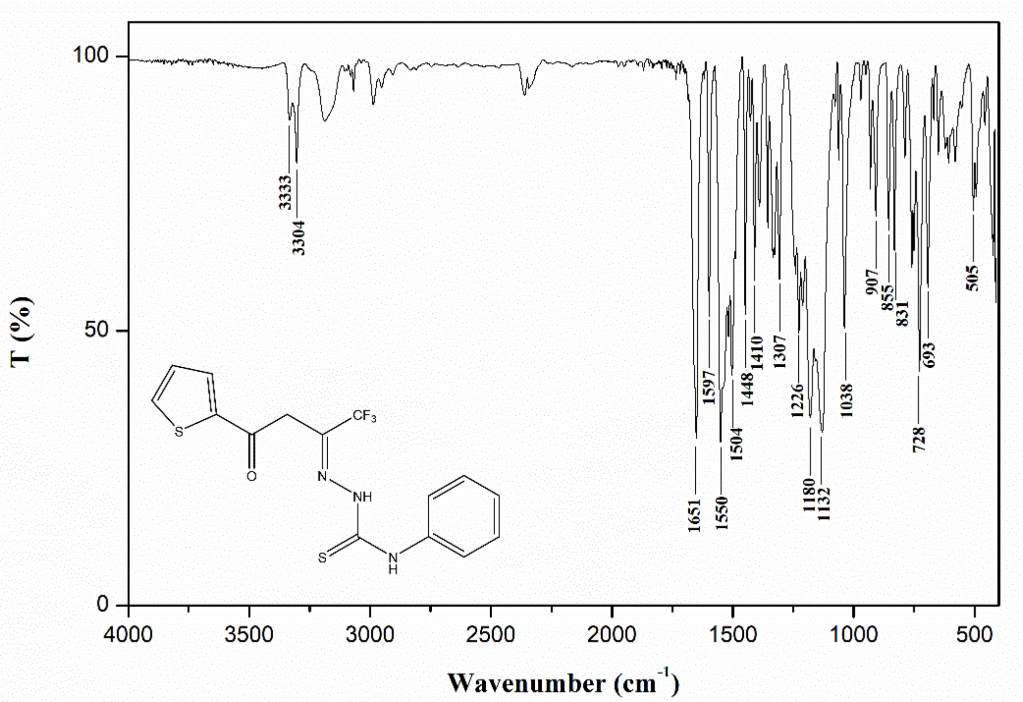

Figure S9. FT-IR spectra of H_2L^1.

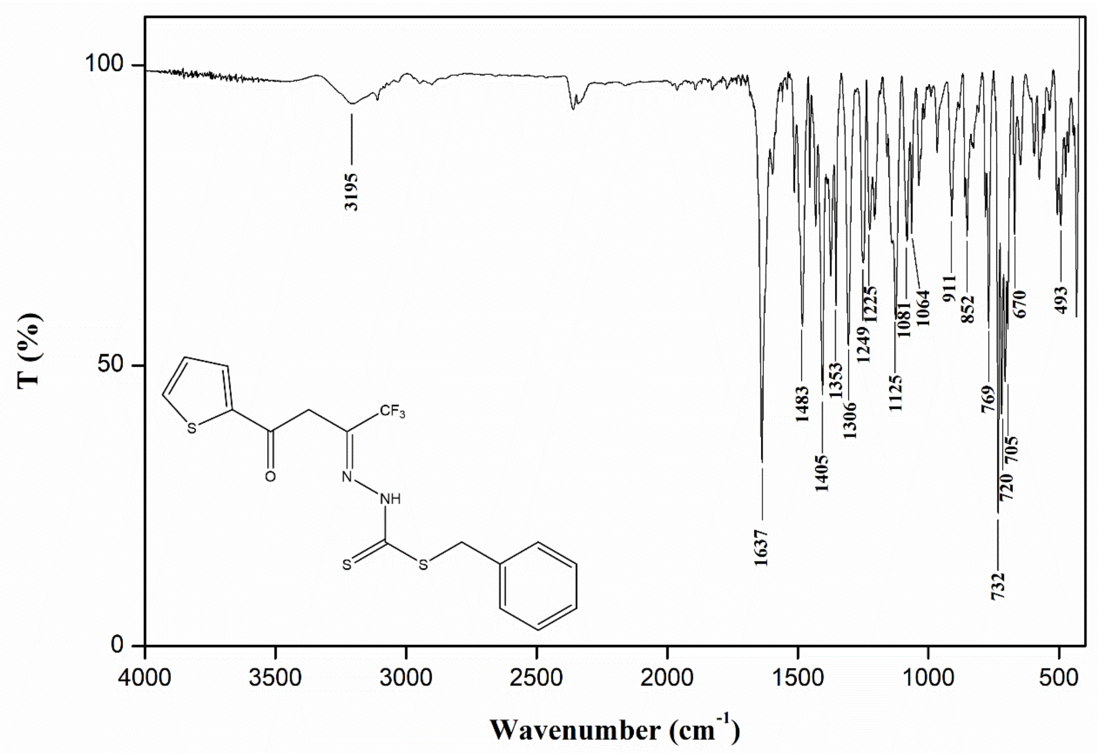

Figure S10. FT-IR spectra of H_2L^2.

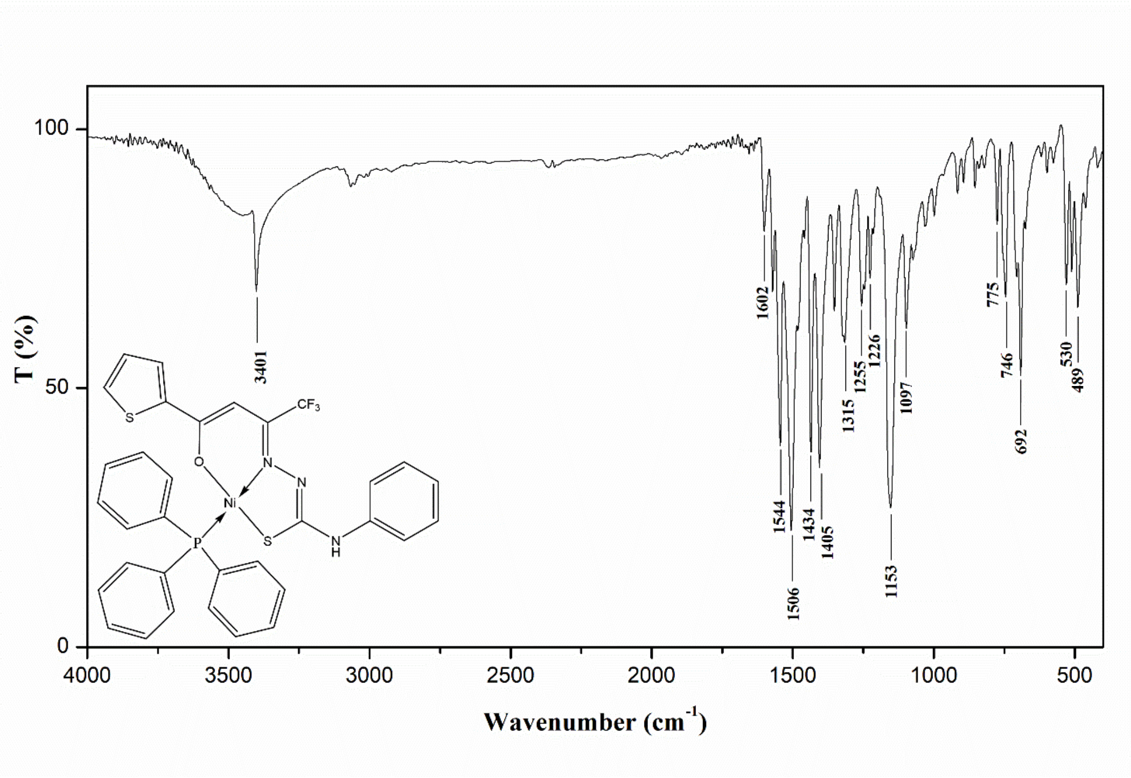

Figure S11. FT-IR spectra of (**1**).

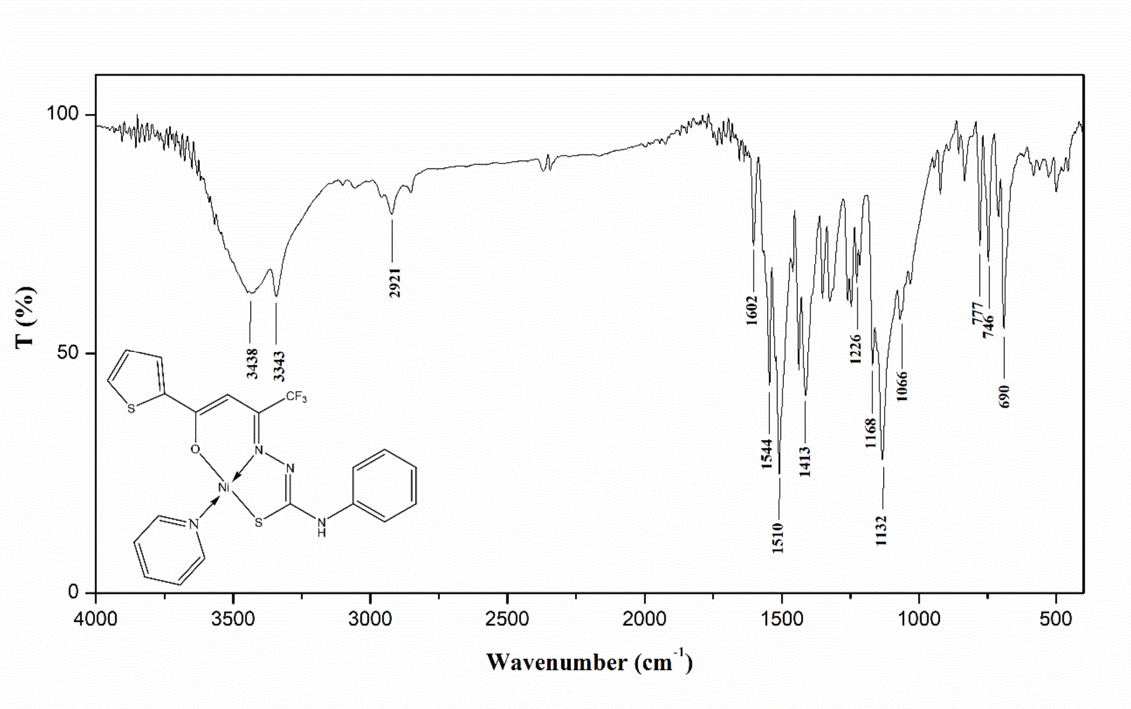

Figure S12. FT-IR spectra of (**2**).

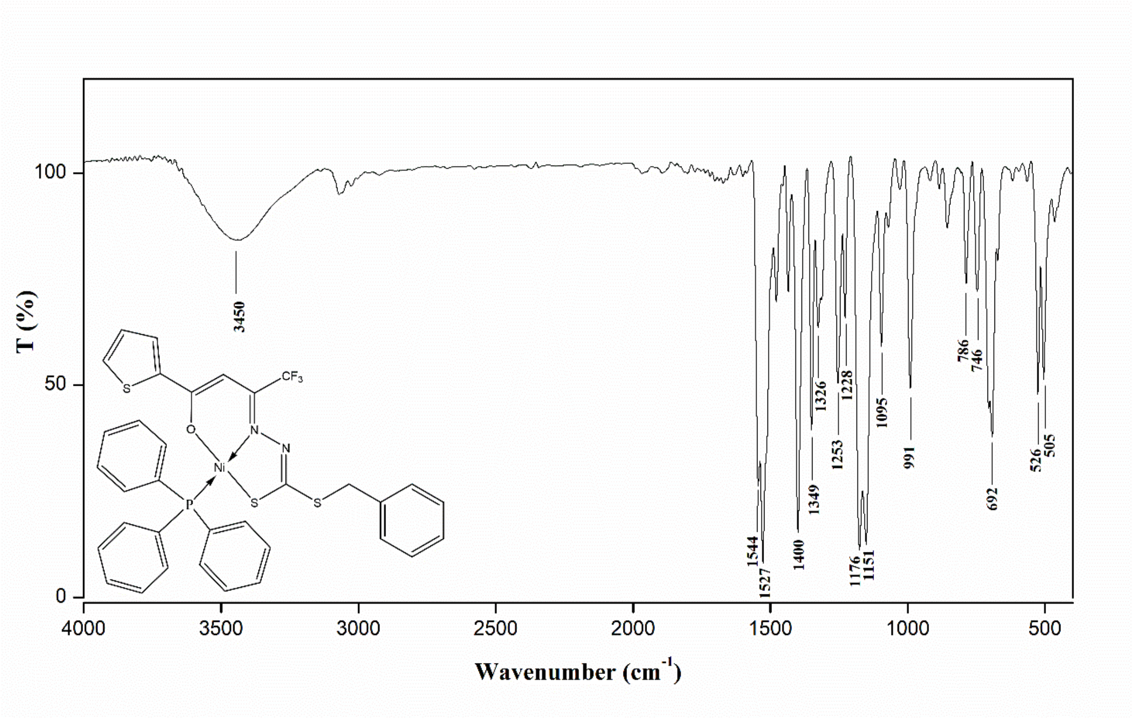

Figure S13. FT-IR spectra of (**3**).

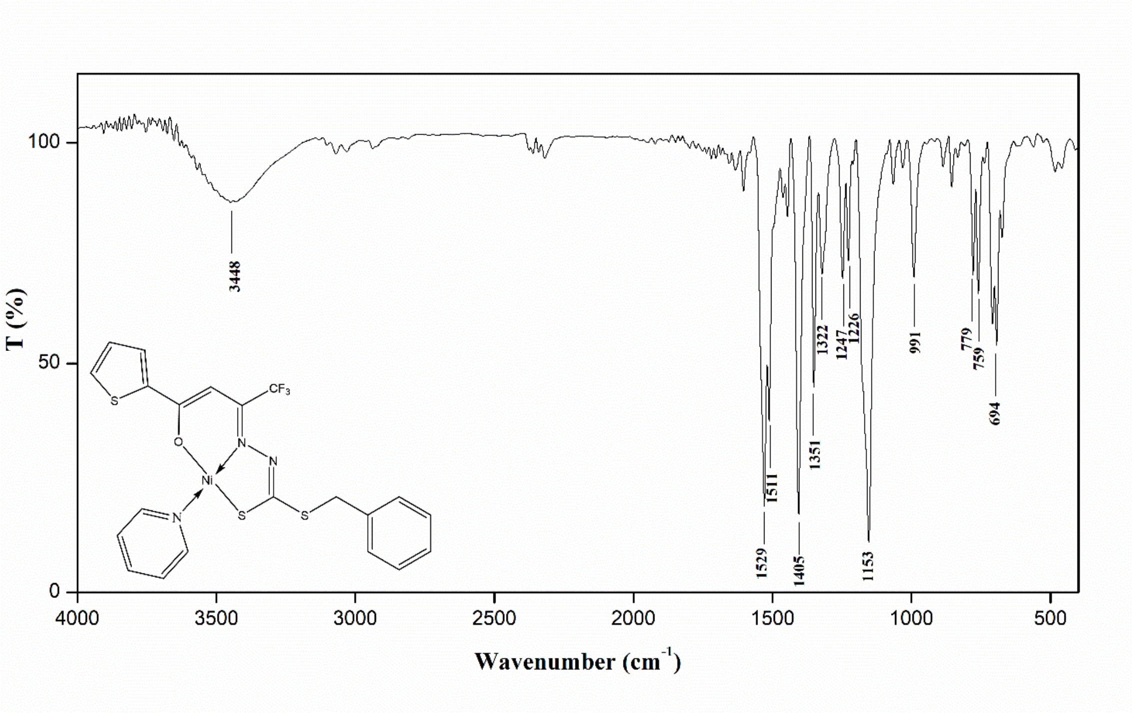

Figure S14. FT-IR spectra of (**4**).

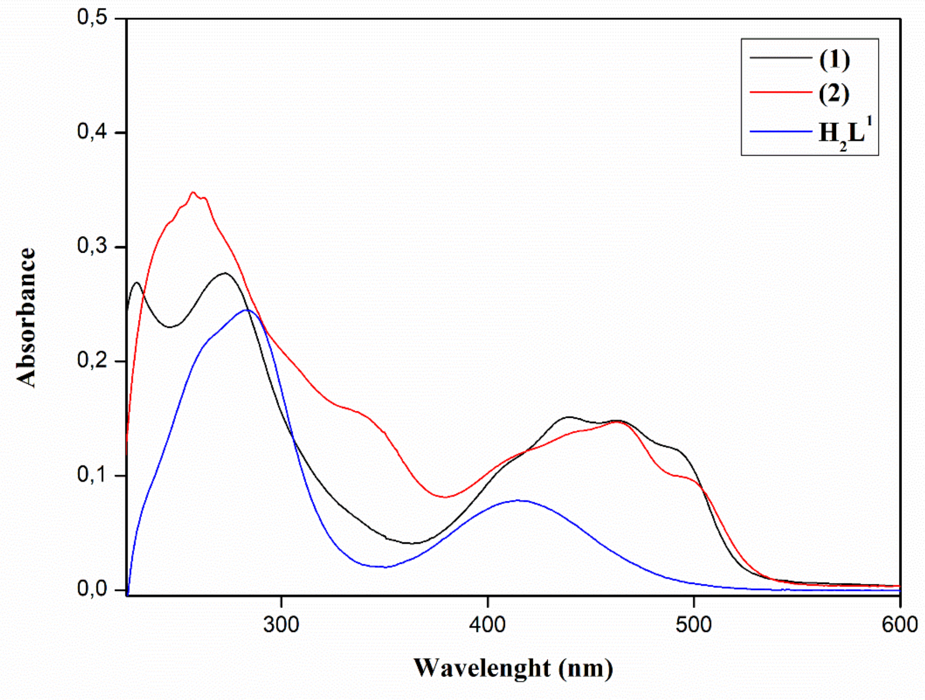

Figure S15. UV-vis spectra of compounds H_2L^1, **(1)** and **(2)** in MeOH.

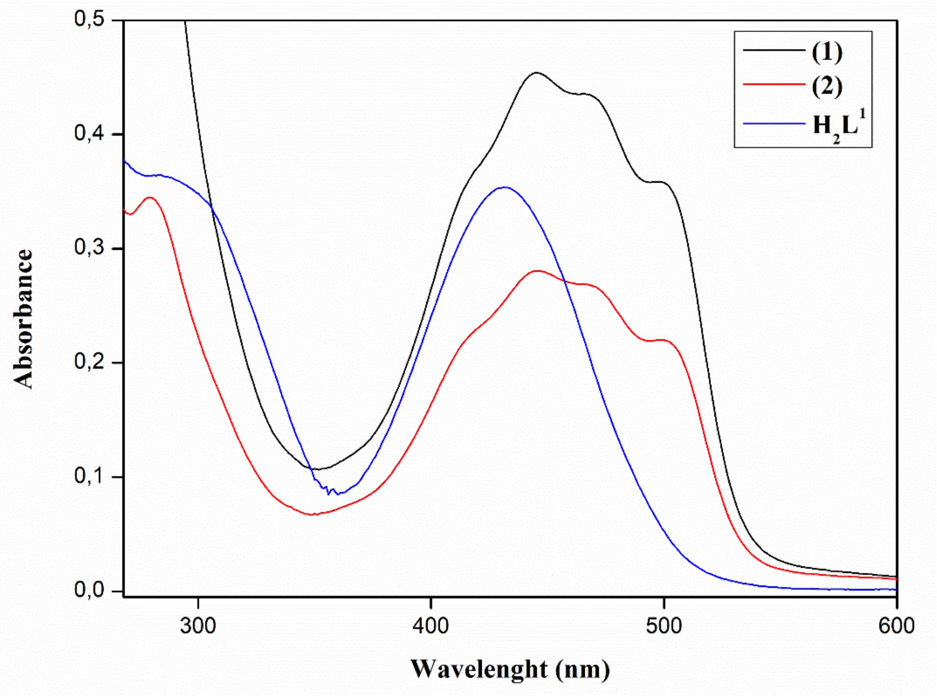

Figure S16. UV-vis spectra of compounds H_2L^1, **(1)** and **(2)** in DMF.

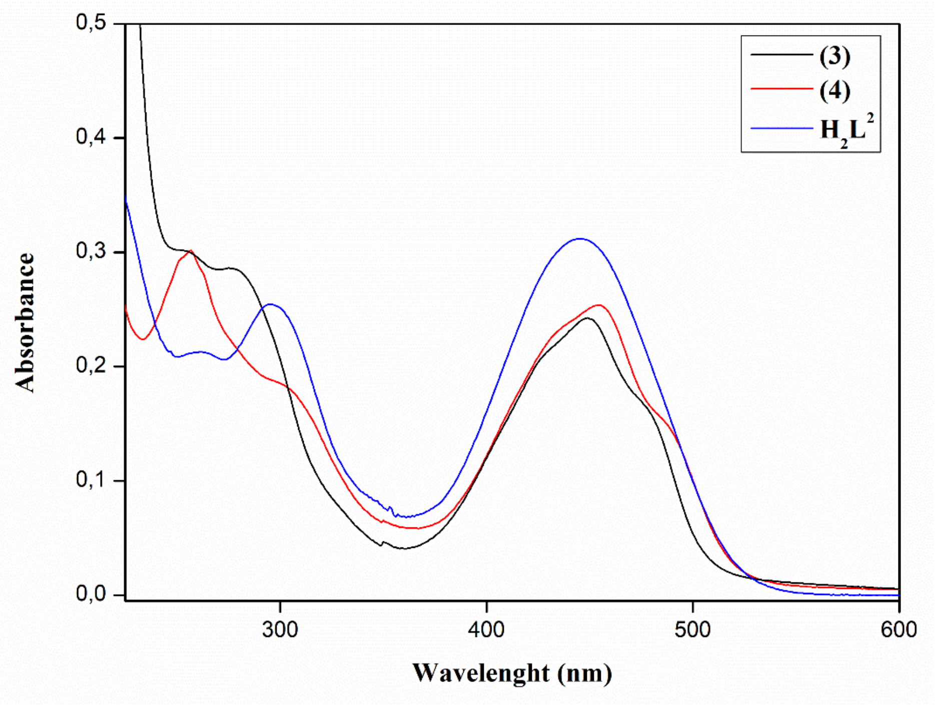

Figure S17. UV-vis spectra of compounds H_2L^2, **(3)** and **(4)** in MeOH.

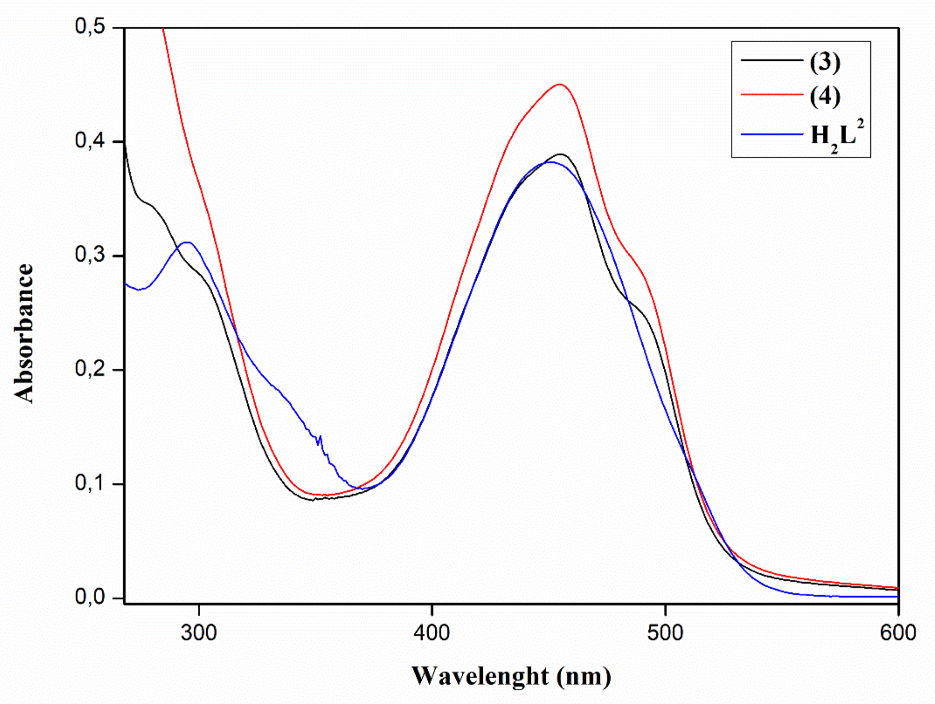

Figure S18. UV-vis spectra of compounds H_2L^2, **(3)** and **(4)** in DMF.

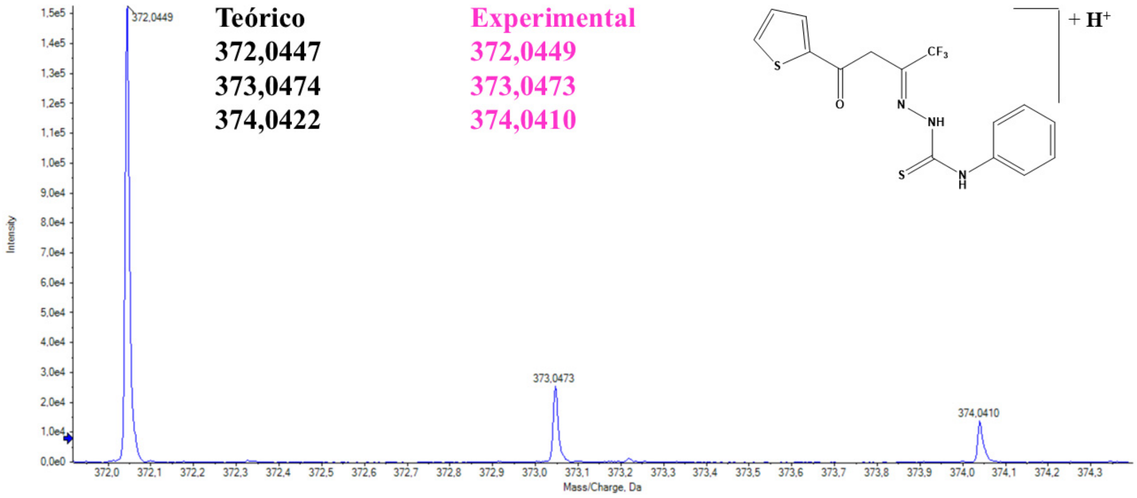

Figure S19. ESI(+)-MS spectrum of H_2L^1.

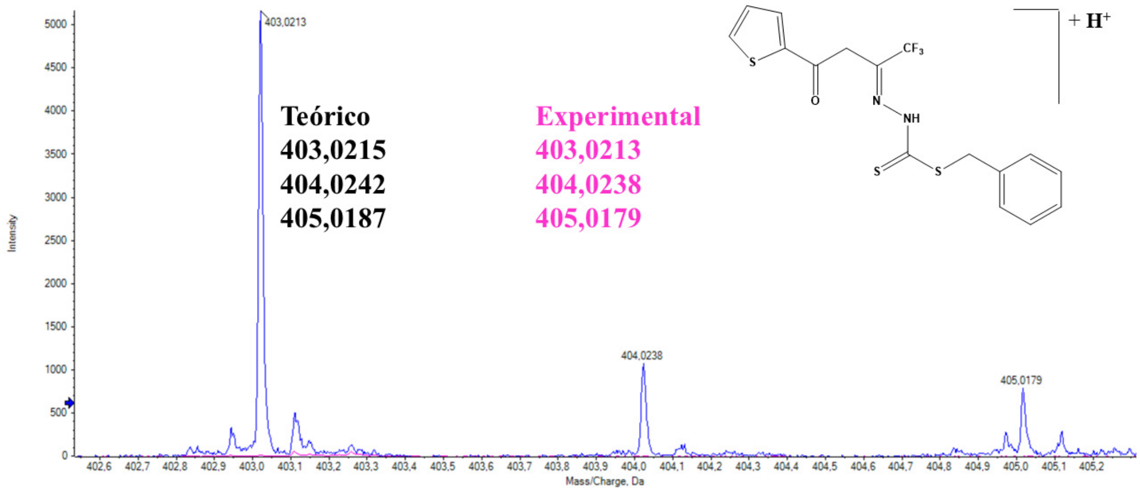

Figure S20. ESI(+)-MS spectrum of H_2L^2.

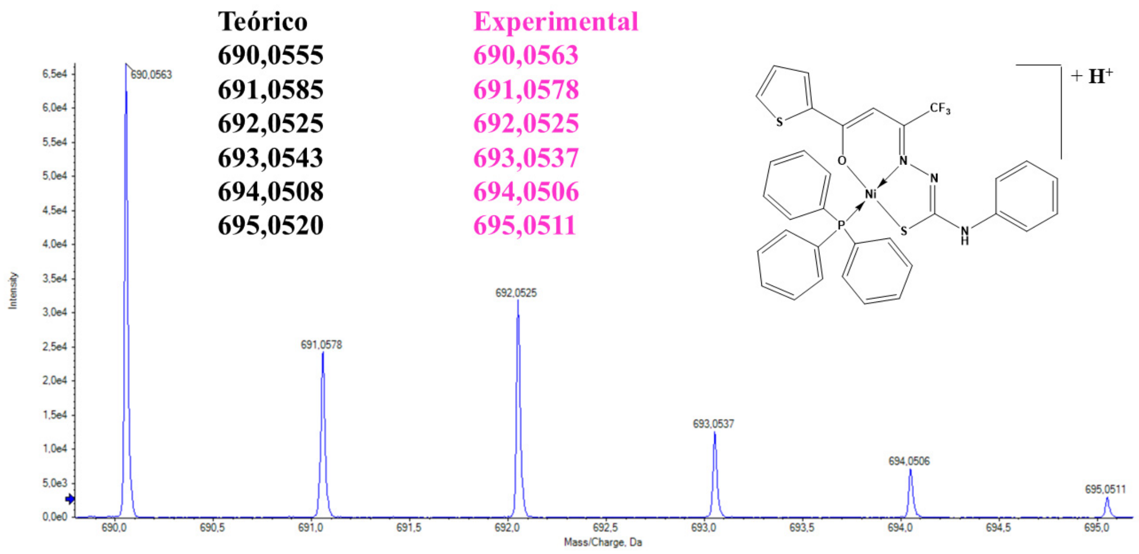

Figure S21. ESI(+)-MS spectrum of **(1)**.

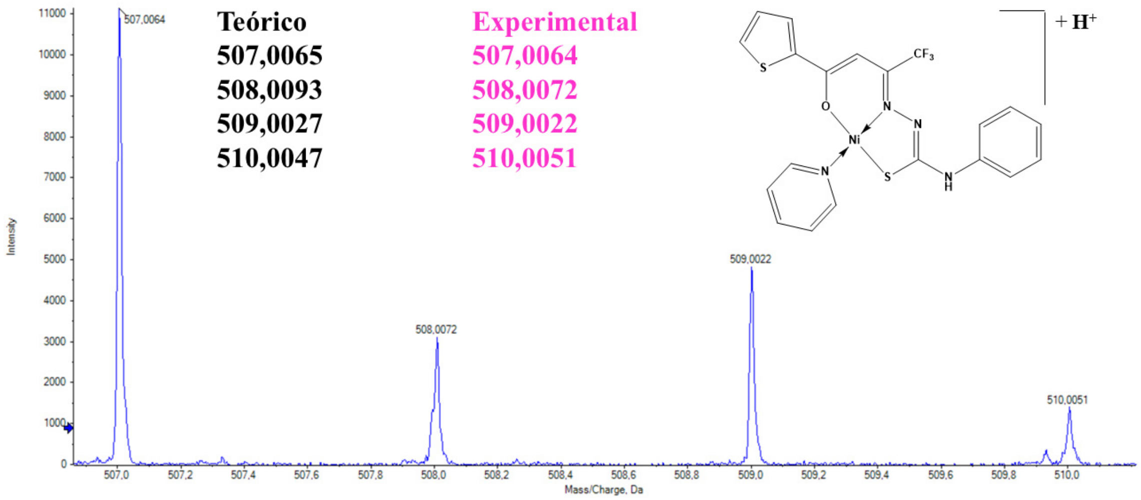

Figure S22. ESI(+)-MS spectrum of **(2)**.

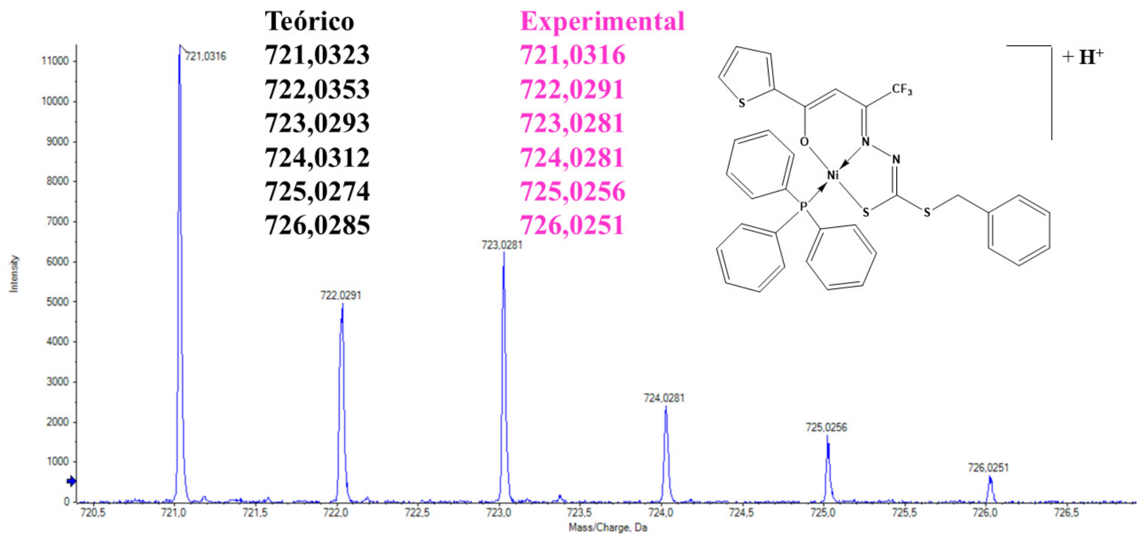

Figure S23. ESI(+)-MS spectrum of **(3)**.

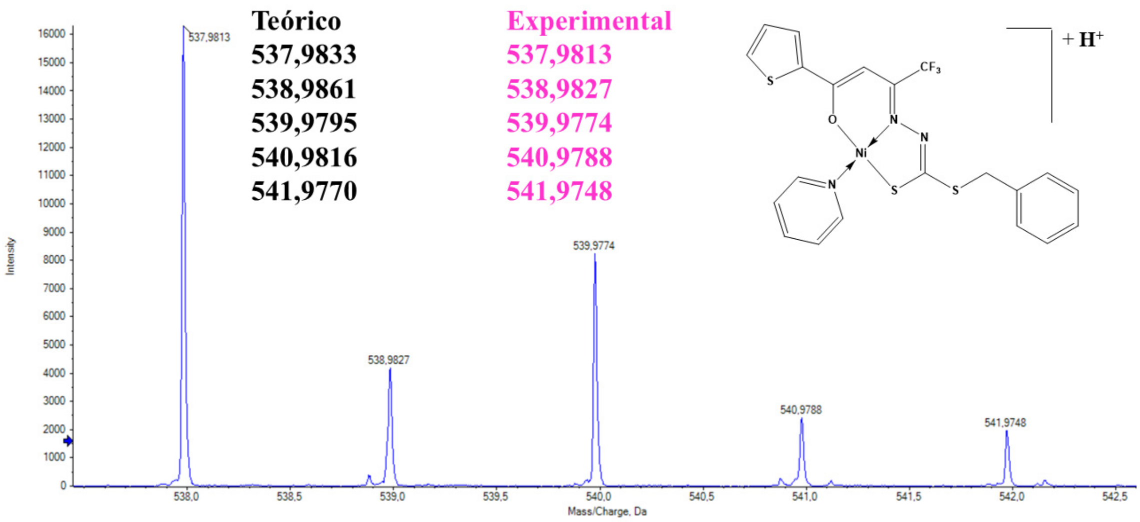

Figure S24. ESI(+)-MS spectrum of **(4)**.

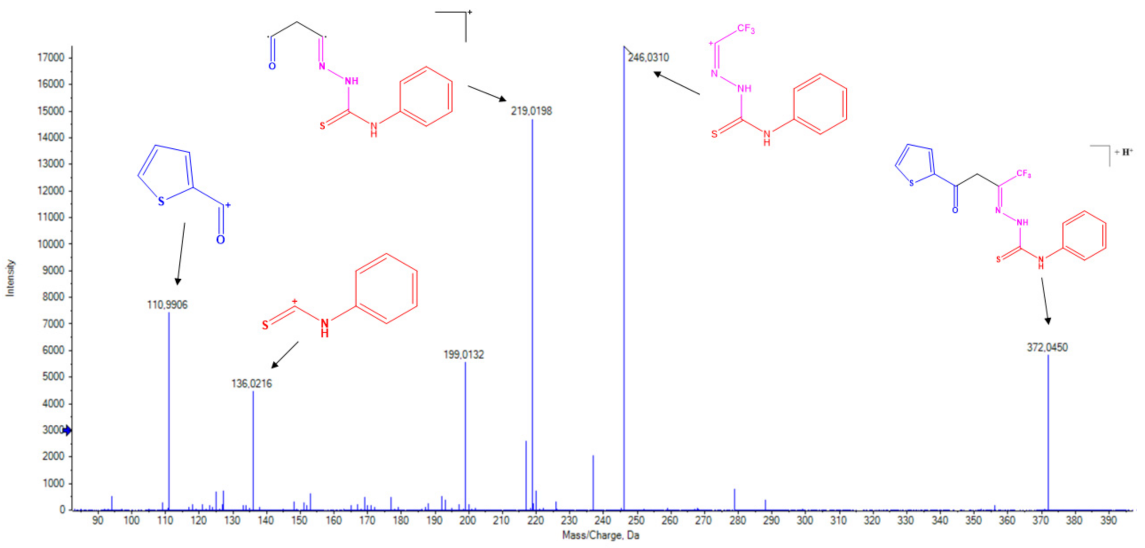

Figure S25. ESI(+)-MS/MS spectrum of H_2L^1.

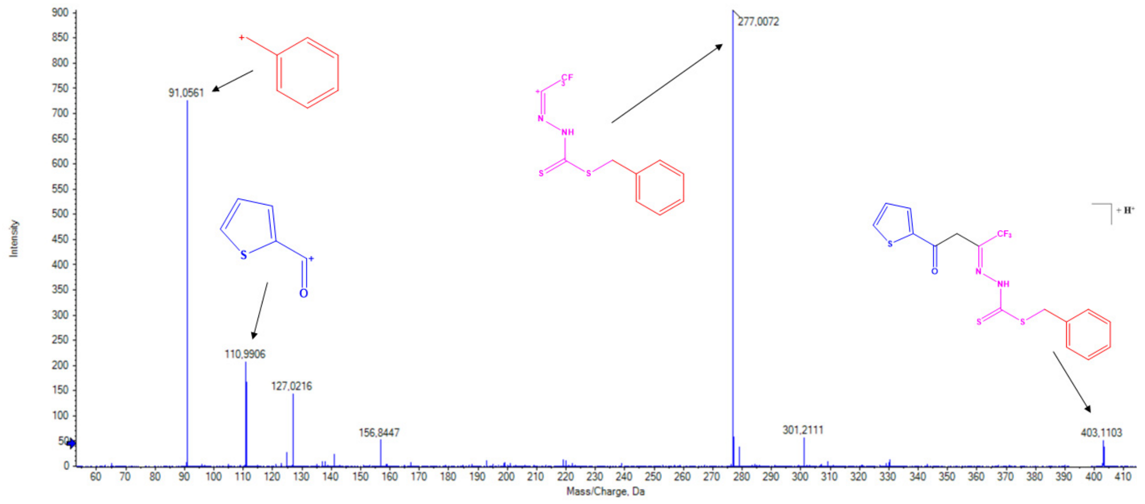

Figure S26. ESI(+)-MS/MS spectrum of H_2L^2.

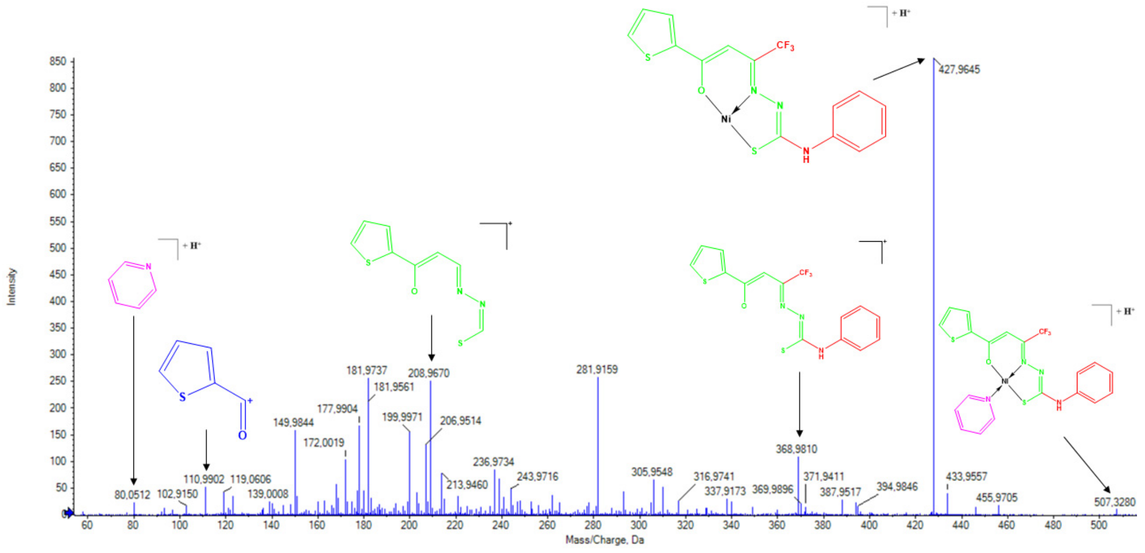

Figure S27. ESI(+)-MS/MS spectrum of **(2)**.

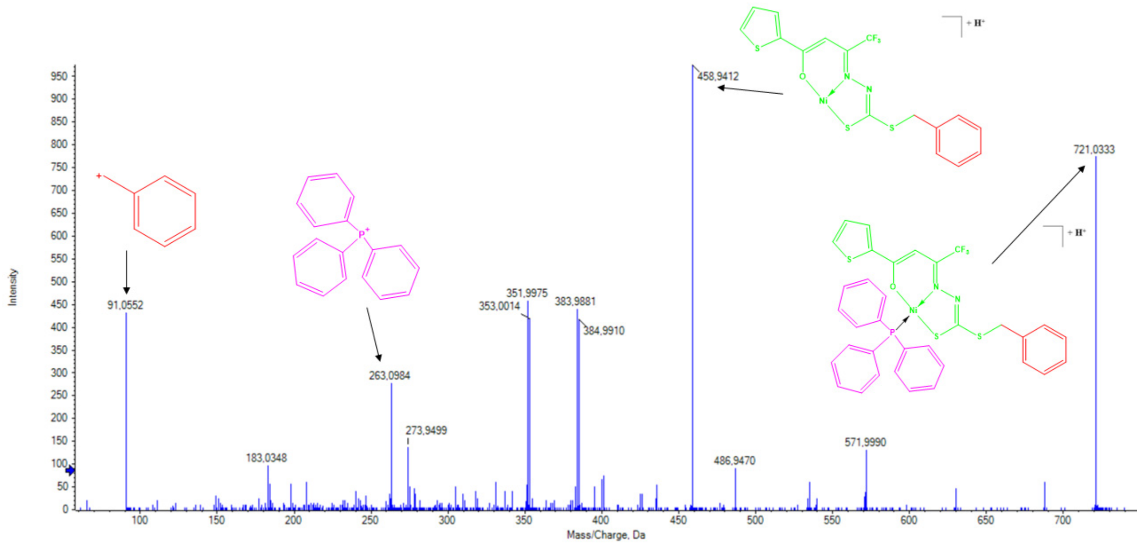

Figure S28. ESI(+)-MS/MS spectrum of **(3)**.

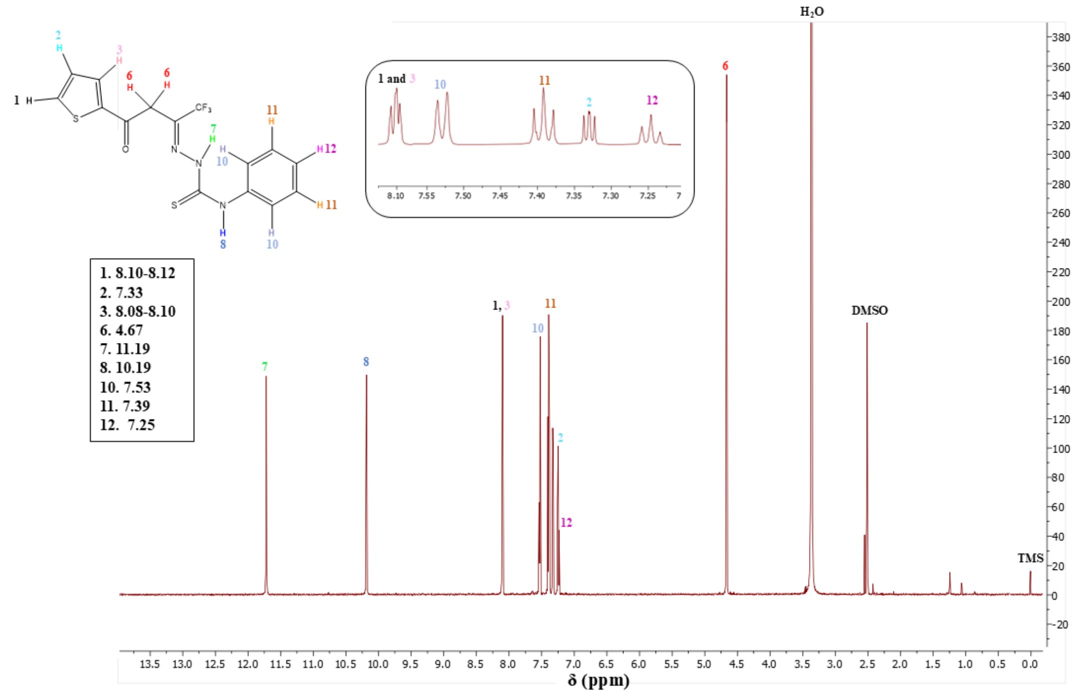

Figure S29. ^{1}H-NMR spectra of H_2L^1.

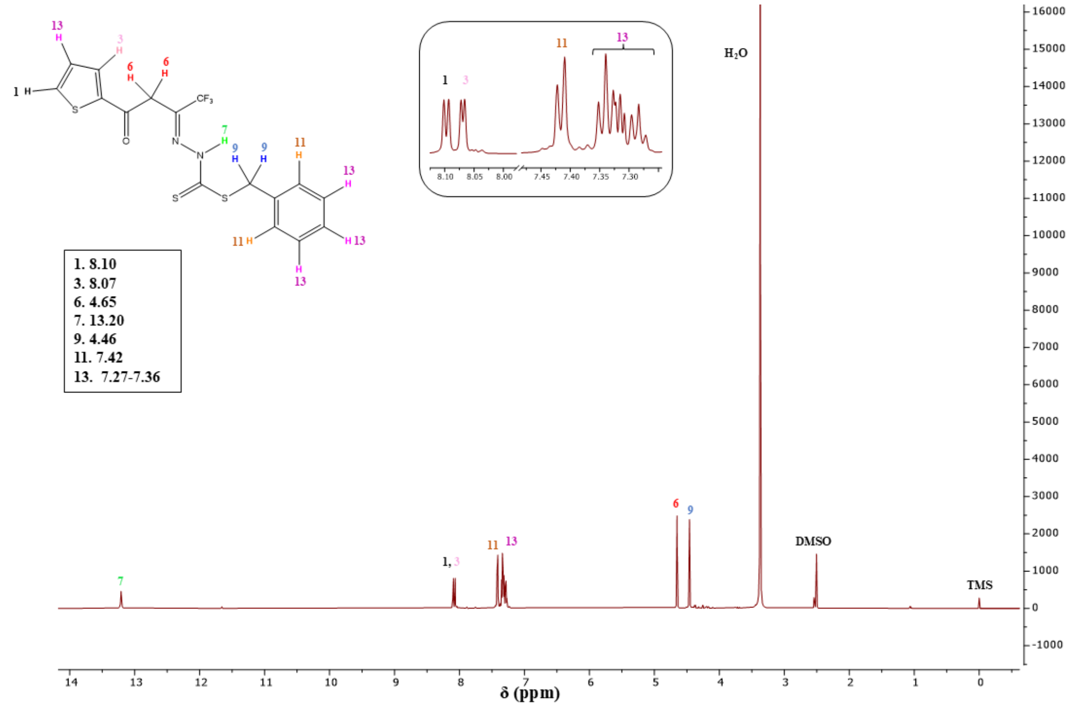

Figure S30. ^{1}H-NMR spectra of H_2L^2.

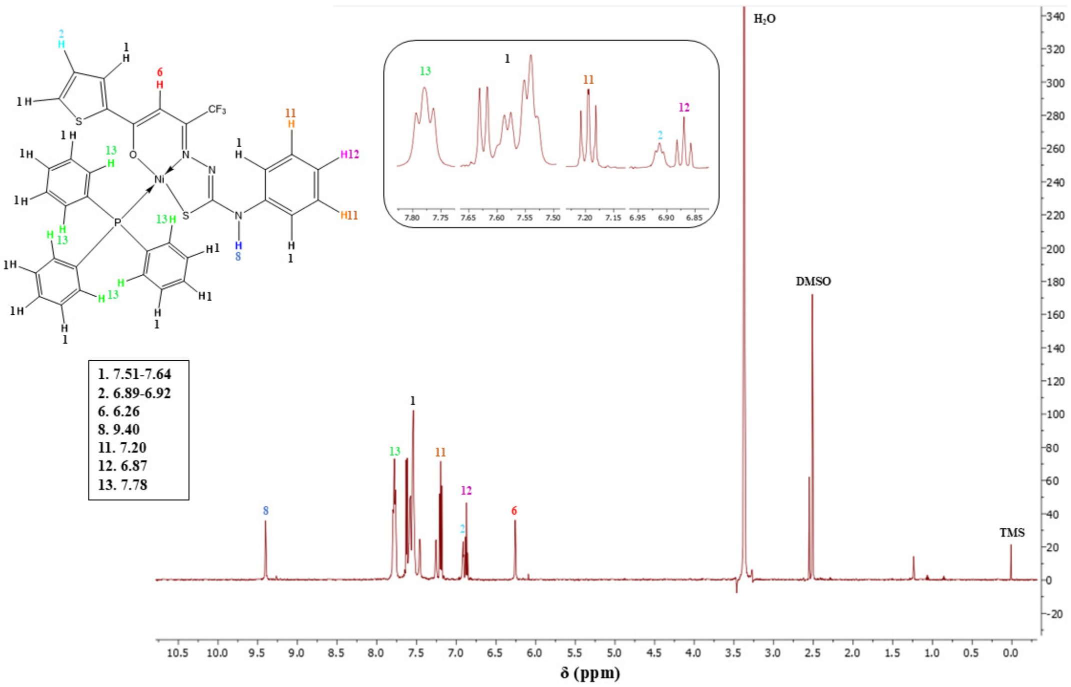

Figure S31. ^{1}H-NMR spectra of **(1)**.

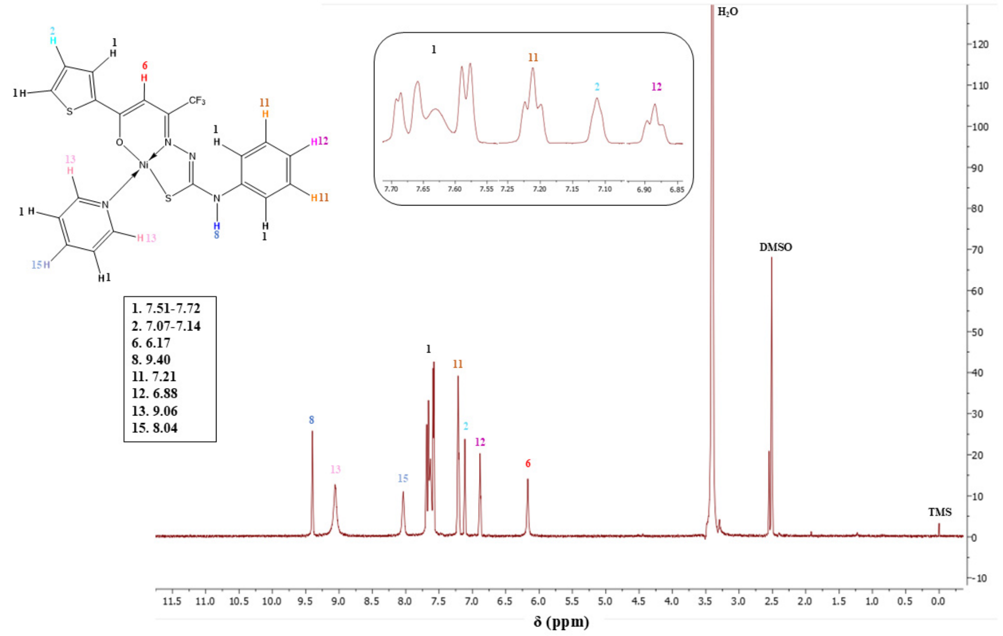

Figure S32. ^{1}H-NMR spectra of **(2)**.

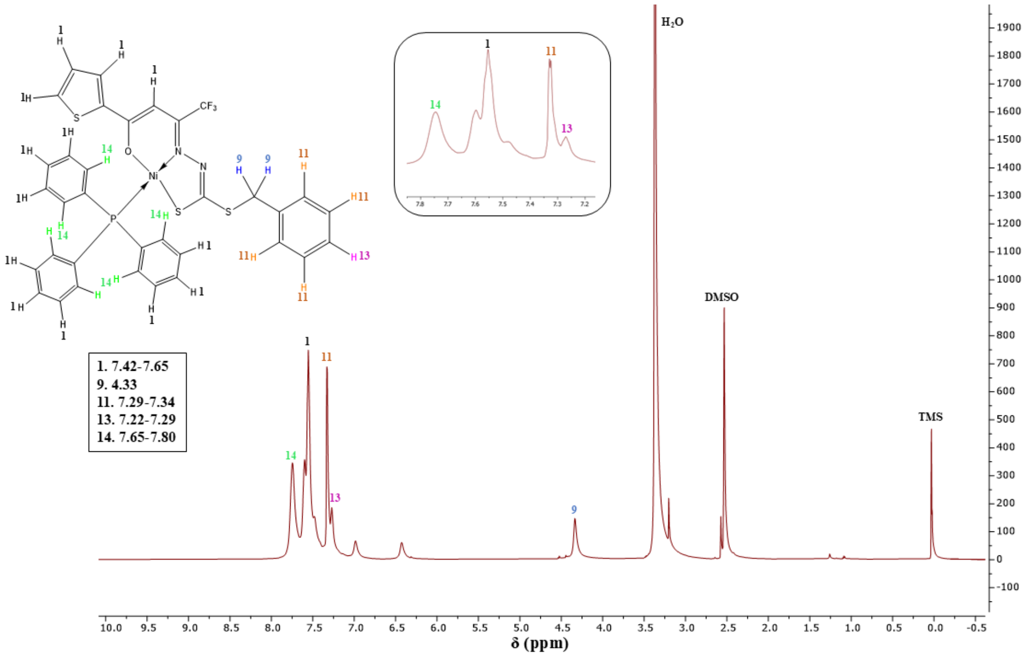

Figure S33. ^{1}H-NMR spectra of **(3)**.

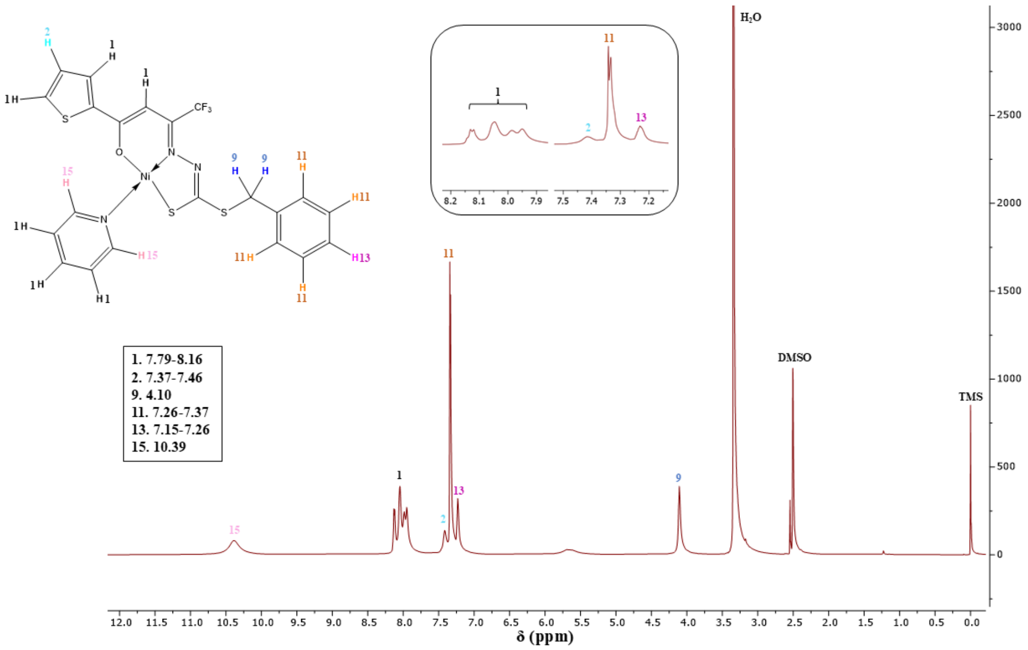

Figure S34. ^{1}H-NMR spectra of **(4)**.

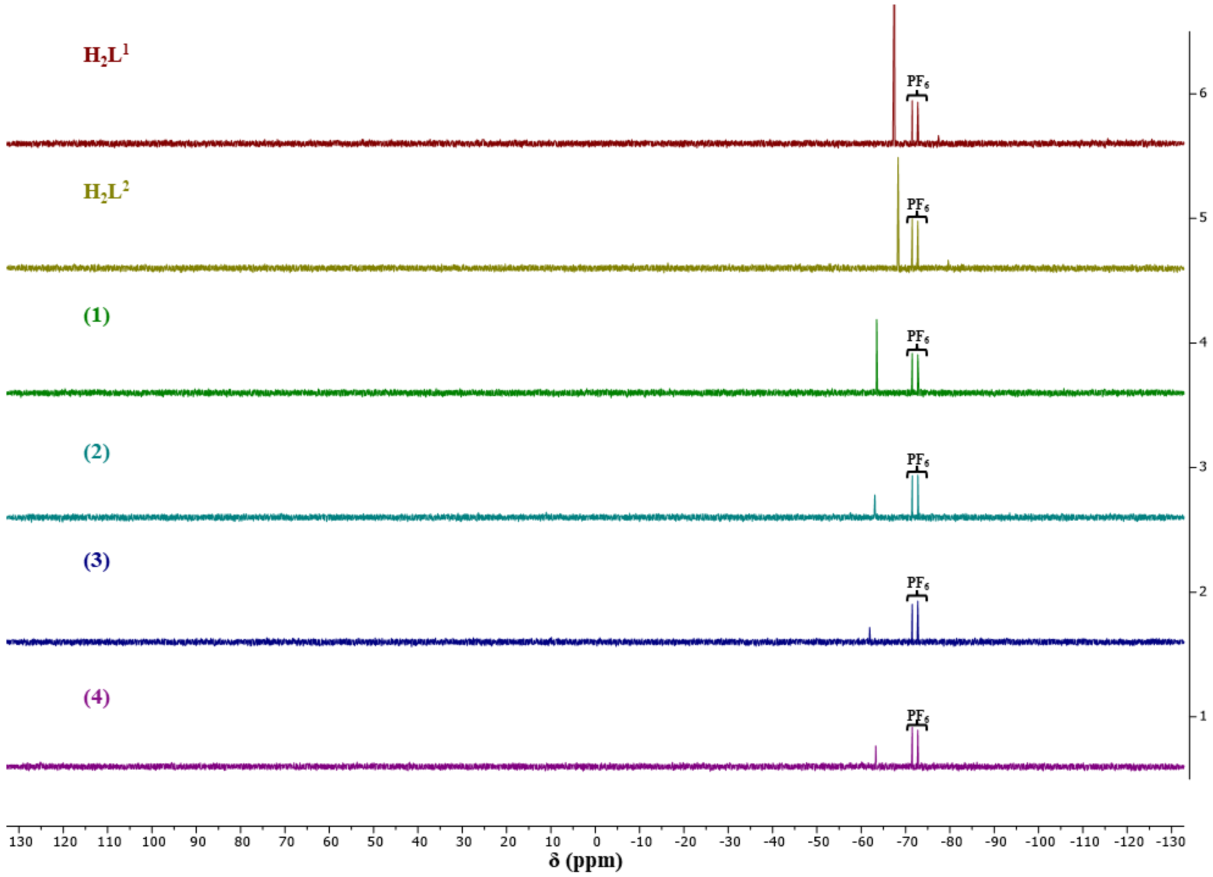

Figure S35. ^{19}F-NMR spectra of ligands H_2L^1 and H_2L^2 and complexes **(1–4)**.

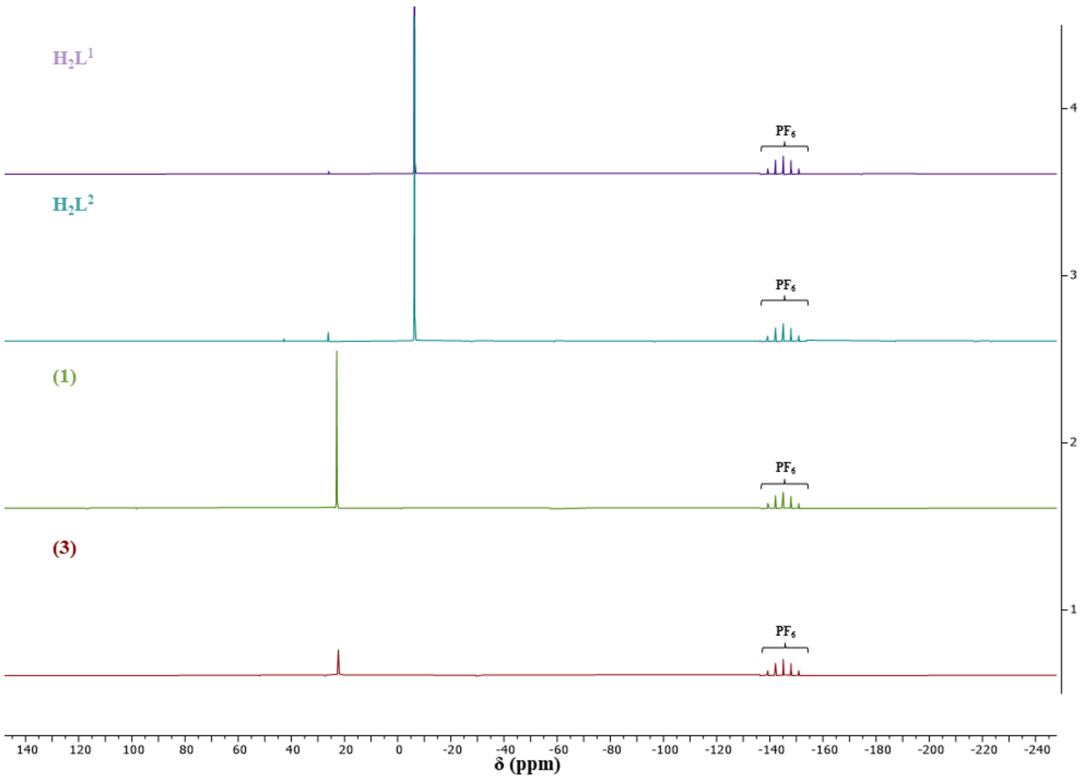

Figure S36. ^{31}P-NMR spectra of free PPh_3 in ligands H_2L^1 and H_2L^2 and complexes **(1)** and **(3)**.

Table S1. Interactions that contribute to the stabilization of the crystal arrangement of complexes **(1-4)**.

Interaction	Distance
**(1)**	
C12-H12···H27	2.878 Å
C2···H21-C21	2.381 Å
**(2)**	
C10···H15-C15	2.887 Å
C9···H15-C15	2.837 Å
N2···H15-C15	2.744 Å
S2···H3-N3	2.987 Å
Ni1···S2	3.254 Å
**(3)**	
C32···H22-C22	2.809 Å
S2···H13-C13	2.589 Å
F1···H15-C15	2.996 Å
**(4)**	
S2···H21-C21	2.883 Å
S2···H20-C20	2.970 Å
N2···H16-C16	2.664 Å

Table S2. ^{1}H-NMR spectrum data for H_2L^1.

δ (ppm)	Multiplicities	Integral	*J* (Hz)	Attribution
4.67	s	2	-	6 (C**H**$_2$)
7.25	t	1	$^{3}J_{H12\text{-}H11}$ = 7.40 Hz	12 (–C**H**$^{12}_{Ar}$)
7.33	dd	1	$^{3}J_{H2\text{-}H1}$ and $^{3}J_{H2\text{-}H3}$ = 3.89 and 3.74 Hz	2 (C**H**)
7.39	dd	1	$^{3}J_{H12\text{-}H11}$ = 7.40 Hz<br>$^{3}J_{H11\text{-}H10}$ = 7.20 Hz	11 (–C**H**$^{11}_{Ar}$)
7.53	d	1	$^{3}J_{H11\text{-}H10}$ = 7.20 Hz	10 (–C**H**$^{10}_{Ar}$)
8.08-8.10	m	1	-	3 (–C**H**)
8.10-8.12	m	1	-	1 (–C**H**)
10.19	s	1	-	8 (–N-**H**)
11.72	s	1	-	7 (–N-**H**)

Table S3. ^{1}H-NMR spectrum data for H_2L^2.

δ (ppm)	Multiplicities	Integral	*J* (Hz)	Attribution
4.46	s	2	-	9 (C**H₂**)
4.65	s	2	-	6 (C**H₂**)
7.27-7.36	m	4	-	13 (–C**H**$^{13}_{Ar}$)
7.42	d	2	$^3J_{H11-H13}$ = 7.50 Hz	11 (–C**H**$^{11}_{Ar}$)
8.07	d	1	$^3J_{H3-H13}$ = 3.80 Hz	3 (–C**H**)
8.10	d	1	$^3J_{H1-H13}$ = 4.92 Hz	1 (–C**H**)
13.20	s	1	-	7 (–N-**H**)

Table S4. ^{1}H-NMR spectrum data for **(1).**

δ (ppm)	Multiplicities	Integral	*J* (Hz)	Attribution
6.26	s	1	-	6 (C**H**)
6.87	t	1	$^3J_{H12-H11}$ = 7.35 Hz	12 (–C**H**$^{12}_{Ar}$)
6.89-6.92	m	1	-	2 (C**H**)
7.20	t	2	-	11 (–C**H**$^{11}_{Ar}$)
7.51-7.64	m	13	-	1 (–C**H**$_{Ar}$)
7.78	t	6	-	13 (–C**H**$^{15}_{Ar}$)

Table S5. ^{1}H-NMR spectrum data for **(2).**

δ (ppm)	Multiplicities	Integral	*J* (Hz)	Attribution
6.17	s	1	-	6 (C**H**)
6.88	t	1	$^3J_{H12-H11}$ = 7.31 Hz	12 (–C**H**$^{12}_{Ar}$)
7.07-7.14	m	1	-	2 (C**H**)
7.21	t	2	$^3J_{H12-H11}$ = 7.31 Hz	11 (–C**H**$^{11}_{Ar}$)
7.51-7.72	m	6	-	1 (–C**H**$_{Ar}$)
8.04	s	1	-	15 (–C**H**$^{15}_{Ar}$)
9.06	s	2	-	13 (–C**H**$^{13}_{Ar}$)
9.40	s	1	-	8 (–N-**H**)

Table S6. ^{1}H-NMR spectrum data for **(3).**

δ (ppm)	Multiplicities	Integral	*J* (Hz)	Attribution
4.33	s	2	-	9 (CH_2)
7.22-7.29	m	1	-	13 (–CH^{13}_{Ar})
7.29-7-34	m	4	-	11 (CH^{11}_{Ar})
7.42-7.65	m	13	-	1 (–CH^{1}_{Ar})
7.65-7.80	m	6	-	14 (–CH_{Ar}=)

Table S7. ^{1}H-NMR spectrum data for **(4).**

δ (ppm)	Multiplicities	Integral	*J* (Hz)	Attribution
4.10	s	2	-	9 (CH_2)
7.15-7.26	m	1	-	13 (–CH^{13}_{Ar})
7.26-7.37	m	4	-	11 (CH^{11}_{Ar})
7.37-7.46	m	1	-	2 (CH)
7.79-8.16	m	13	-	1 (–CH^{1}_{Ar})
10.39	s	2	-	15 (–CH^{13}_{Ar})

Table S8. Molar conductivity data ($\Omega^{-1}.cm^2.mol^{-1}$) at 0, 24 and 48 hours of DMSO and complexes (1–4).

**Compound**	**0 hours**	**24 hours**	**48 hours**
**DMSO**	1.57	1.55	1.74
**(1)**	1.64	2.28	3.21
**(2)**	1.73	3.69	5.54
**(3)**	1.87	2.29	2.37
**(4)**	2.23	3.22	4.27

Table S9. X-ray diffraction data collection and refinement parameters for the complexes **(1-4).**

	**(1)**	**(2)**	**(3)**	**(4)**
Chemical formula	$C_{33}H_{25}F_3N_3OS_2PNi$	$C_{20}H_{15}F_3N_4OS_2Ni$	$C_{34}H_{26}F_3N_2OPS_3Ni$	$C_{21}H_{16}F_3N_3OS_3Ni$
M (g mol^{-1})	690.36	507.18	721.43	538.26
Crystal system	Triclinic	Trigonal	Triclinic	Triclinic
Space group	P–1	R3c	P–1	P–1
Unit cell				
a (Å)	10.342(12)	38.253(8)	9.425(3)	6.559(19)
b (Å)	12.001(15)	38.253(8)	11.480(4)	10.411(3)
c (Å)	13.975(17)	7.5078(2)	16.706(5)	16.696(5)
α (º)	75.036(6)	90	91.514(7)	88.948(6)
β (º)	70.576(5)	90	98.188(6)	86.683(6)
γ (º)	77.095(5)	120	113.667(6)	88.745(6)
V (Å^3)	1562.1(3)	9514(5)	1631.7(9)	1137.8(6)
Z	2	18	2	2
Density (g cm^{-3})	1.468	1.593	1.468	1.571
Index ranges	$-13 \leq h \leq 13$, $-15 \leq k \leq 15$, $-18 \leq l \leq 18$	$-46 \leq h \leq 45$, $-46 \leq k \leq 46$, $-9 \leq l \leq 9$	$-11 \leq h \leq 11$, $-13 \leq k \leq 13$, $-20 \leq l \leq 20$	$-7 \leq h \leq 7$, $-12 \leq k \leq 12$, $-20 \leq l \leq 20$
Absorption coefficient μ (mm^{-1})	0.856	1.161	0.884	1.171
Absorption correction	Multi-scan	Multi-scan	Multi-scan	Multi-scan
Measured reflections	35013	41071	21419	15302
Independent reflections/Rint	7211/0.106	3890/0.160	5964/0.102	4216/0.107
Refined parameters	399	281	407	290
R_1 (F)	0.0516	0.0471	0.0542	0.0595
wR_2 (F^2) ($I > 2\sigma(I)$)	0.0988	0.0854	0.1193	0.1358
Largest diff. peak and hole (eÅ^{-3})	0.32 and -0.36	0.23 and -0.25	0.40 and -0.67	0.46 and - 0.43
CCDC Nº	2423757	2423758	2423759	2423760
